# Age could be driving variable SARS-CoV-2 epidemic trajectories worldwide

**DOI:** 10.1371/journal.pone.0237959

**Published:** 2020-08-20

**Authors:** Houssein H. Ayoub, Hiam Chemaitelly, Shaheen Seedat, Ghina R. Mumtaz, Monia Makhoul, Laith J. Abu-Raddad

**Affiliations:** 1 Department of Mathematics, Statistics, and Physics, Qatar University, Doha, Qatar; 2 Infectious Disease Epidemiology Group, Weill Cornell Medicine-Qatar, Cornell University, Qatar Foundation – Education City, Doha, Qatar; 3 World Health Organization Collaborating Centre for Disease Epidemiology Analytics on HIV/AIDS, Sexually Transmitted Infections, and Viral Hepatitis, Weill Cornell Medicine – Qatar, Cornell University, Qatar Foundation – Education City, Doha, Qatar; 4 Department of Population Health Sciences, Weill Cornell Medicine, Cornell University, New York City, New York, United States of America; 5 Department of Epidemiology and Population Health, American University of Beirut, Beirut, Lebanon; University of KwaZulu-Natal School of Social Sciences, SOUTH AFRICA

## Abstract

Current geographic spread of documented severe acute respiratory syndrome coronavirus 2 (SARS-CoV-2) infections shows heterogeneity. This study explores the role of age in potentially driving differentials in infection spread, epidemic potential, and rates of disease severity and mortality across countries. An age-stratified deterministic mathematical model that describes SARS-CoV-2 transmission dynamics was applied to 159 countries and territories with a population ≥1 million. Assuming worst-case scenario for the pandemic, the results indicate that there could be stark regional differences in epidemic trajectories driven by differences in the distribution of the population by age. In the African Region (median age: 18.9 years), the median *R*_0_ was 1.05 versus 2.05 in the European Region (median age: 41.7 years), and the median (per 100 persons) for the final cumulative infection incidence was 22.5 (versus 69.0), for severe and/or critical disease cases rate was 3.3 (versus 13.0), and for death rate was 0.5 (versus 3.9). Age could be a driver of variable SARS-CoV-2 epidemic trajectories worldwide. Countries with sizable adult and/or elderly populations and smaller children populations may experience large and rapid epidemics in absence of interventions. Meanwhile, countries with predominantly younger age cohorts may experience smaller and slower epidemics. These predictions, however, should not lead to complacency, as the pandemic could still have a heavy toll nearly everywhere.

## Introduction

The current geographic spread of the documented severe acute respiratory syndrome coronavirus 2 (SARS-CoV-2) [1] infections and associated Coronavirus Disease 2019 (COVID-2019) [1] shows heterogeneity [2]. This remains unexplained but may possibly reflect delays in virus introduction into the population, differentials in testing and/or reporting, differentials in the implementation, scale, adherence, and timing of public health interventions, or other epidemiological factors. In this study, we explore the role of age in explaining the differential spread of the infection and its future epidemic potential.

As our understanding of the SARS-CoV-2 transmission dynamics is rapidly evolving [3], the role of age in the epidemiology of this infection is becoming increasingly apparent [4–7]. In a recent study examining SARS-CoV-2 epidemiology in China [4], we quantified the effect of age on the *biological* susceptibility to infection acquisition. We found that *relative* to individuals aged 60–69 years, susceptibility was 0.06 in those aged 0–19 years, 0.34 in those aged 20–29 years, 0.57 in those aged 30–39 years, 0.69 in those aged 40–49 years, 0.79 in those aged 50–59 years, 0.94 in those aged 70–79 years, and 0.88 in those aged ≥80 years (S1 Fig in S1 File). Notably, this age-dependence was estimated after accounting for the assortativeness in population mixing by age [4].

Age also affects disease progression [7–10] and mortality risk [11–13] among those infected. The proportion of infections that eventually progress to severe disease, critical disease, or death, increases rapidly with age, especially among those ≥50 years of age (S2 Fig in S1 File) [8–12]. Since the demographic structure of the population (that is the distribution of the population across the different age groups) varies by country and region, this poses a question as to the extent to which age effects can drive geographic differentials in the basic reproduction number (*R*_0_), epidemic potential, and rates of disease severity and mortality. *R*_0_ is defined here as the number of infections caused by an index case in a fully susceptible population [14].

We aimed here to provide an answer to this question and to estimate for each country (with a population ≥1 million), region, and globally, *R*_0_, and the rate per 100 persons (out of the total population by the end of the epidemic cycle) of each of the cumulative number of incident infections, mild infections, severe and/or critical disease cases, and deaths, in addition to the number of days needed for the national epidemic to reach its incidence peak (a measure of how fast the epidemic will grow).

## Materials and methods

We adapted and applied our recently developed deterministic mathematical model [4] describing SARS-CoV-2 transmission dynamics in China (S3 Fig in S1 File), to 159 countries and territories, virtually covering the world population [15]. Since our focus was on investigating the natural course of the SARS-CoV-2 epidemic and on assessing its full epidemic potential, the model was applied assuming the *worst-case* scenario for the epidemic in each country, that is in absence of any intervention.

The model stratified the population into compartments according to age (0–9, 10–19, …, ≥80 years), infection status (uninfected, infected), infection stage (mild, severe, critical), and disease stage (severe, critical), using a system of coupled nonlinear differential equations (Section 1 in S1 File). Susceptible individuals in each age group were assumed at risk of acquiring the infection at a hazard rate that varies based on the age-specific susceptibility to the infection (S1 Fig in S1 File), the infectious contact rate per day, and the transmission mixing matrix between the different age groups (Section 1 in S1 File). Following a latency period of 3.69 days [16–19], infected individuals develop mild, severe, or critical infection, as informed by the observed age-specific distribution of cases across these infection stages in China [8–10]. The duration of infectiousness was assumed to last for 3.48 days [8, 16, 18, 19] after which individuals with mild infection recover, while those with severe and critical infection develop, respectively, severe and critical disease over a period of 28 days [8] prior to recovery. Individuals with critical disease have the additional risk of disease mortality, as informed by the age-stratified disease mortality rate in China [11, 12].

Model parameters were based on current data for SARS-CoV-2 natural history and epidemiology, or through model fitting to the China outbreak data [4] (S1, S2 Tables in S1 File). Namely, the overall infectious contact rate, age-specific biological susceptibility profile, and age-specific mortality rate (assuming the observed case fatality rate) were assumed as those in China [4]. The population size, demographic structure, and life expectancy in countries and territories with a population of ≥1 million, as of 2020, were extracted from the United Nations World Population Prospects database [15]. Whenever available data for a parameter covered a wider age bracket, we assumed that this parameter has the same value for all age groups within that bracket. Model parameters, definitions, values, and justifications are in Section B and S1, S2 Tables in S1 File.

Ranges of uncertainty around model-predicted outcomes were determined using five-hundred simulation runs that applied Latin Hypercube sampling [20, 21] from a multidimensional distribution of the model parameters, including both the natural history parameters and the age-specific susceptibility profile. At each run, input parameter values were selected from ranges specified by assuming ±30% uncertainty around parameters’ point estimates [22–26]. The resulting distribution for each model-predicted outcome was then used to derive the most probable estimate (based on maximum likelihood) and 95% uncertainty interval. Mathematical modelling analyses were performed in MATLAB R2019a [27], while statistical analyses were performed in STATA/SE 16.1 [28].

## Results

**Fig 1:**
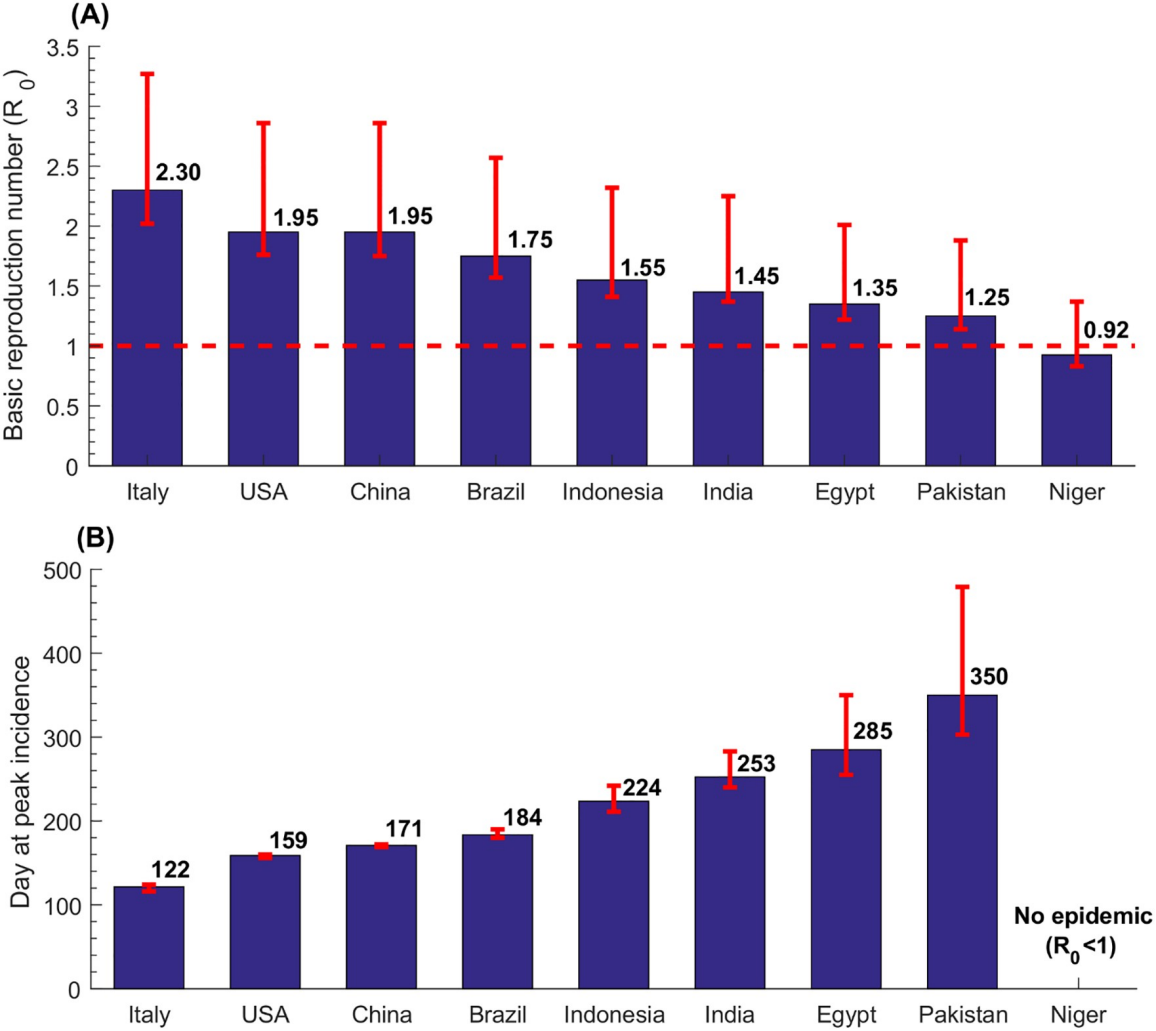
Estimates for the basic reproduction number, *R*_0_, and the number of days needed for the national epidemic to reach its incidence peak, in select countries.

In what follows, we highlight results for select (mostly populous) countries that are of broad geographic representation and characterized by diverse demographic structures. These include Brazil, China, Egypt, India, Indonesia, Italy, Niger, Pakistan, and USA. Estimates and uncertainty ranges for these countries are illustrated in Figs 1, 3 and 4. Detailed results for all 159 countries and territories are in S3-S8 Tables in S1 File.

**Fig 2:**
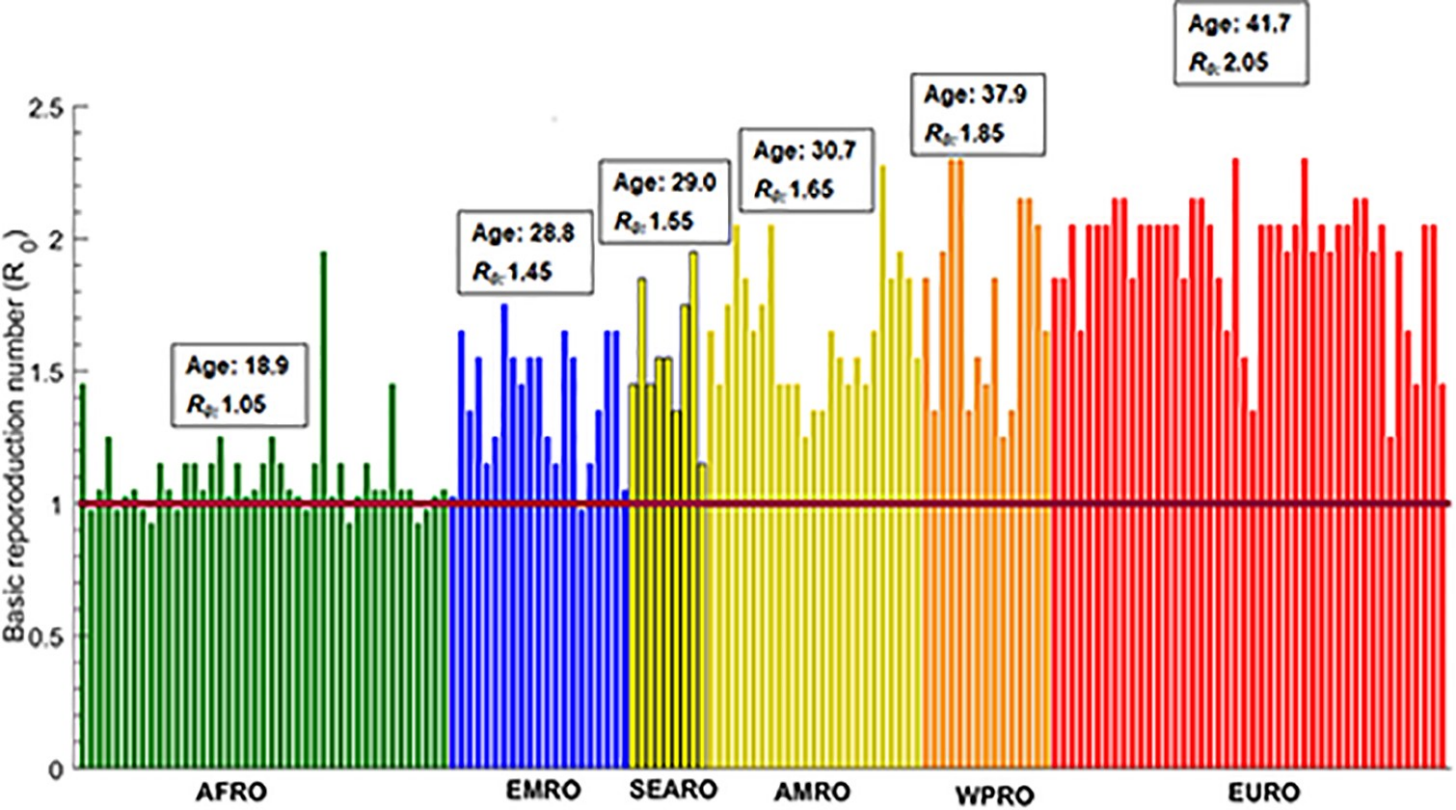
Estimates for the basic reproduction number *R*_0_ in 159 countries and territories with a population of at least one million, across World Health Organization regions. These are the African Region (AFRO), Eastern Mediterranean Region (EMRO), South-East Asia Region (SEARO), Region of the Americas (AMRO), Western Pacific Region (WPRO), and European Region (EURO). The figure shows also the median age in years and the median *R*_0_ for each world region.

Fig 1A shows the estimated *R*_0_ in these select countries, which was highest in Italy at 2.30, followed by USA and China at 1.95, Brazil at 1.75, Indonesia at 1.55, India at 1.45, Egypt at 1.35, Pakistan at 1.25, and lowest in Niger at 0.93. Country-specific estimates of *R*_0_ grouped by World Health Organization (WHO) region are illustrated in Fig 2. The median *R*_0_ was 1.05 (range: 0.93–1.95) in African Region (AFRO), 1.45 (range: 0.98–1.75) in Eastern Mediterranean Region (EMRO), 1.55 (range: 1.15–1.95) in South-East Asia Region (SEARO), 1.65 (range: 1.25–2.28) in Region of the Americas (AMRO), 1.85 (range: 1.25–2.30) in Western pacific Region (WPRO), and 2.05 (range: 1.25–2.30) in European Region (EURO). Globally, the median *R*_0_ was 1.55 with a range of 0.93–2.30.

Fig 1B shows the estimated number of days needed for the national epidemic to reach its incidence peak in the select countries. In Italy, the peak was reached after 122 days (~4 months), whereas in Pakistan it was reached after 350 days (nearly a year). Meanwhile, since *R*_0_<1, no epidemic would emerge in Niger. Of note that the number of days needed for the national epidemic to reach its incidence peak depends on the population size in addition to *R*_0_—it takes more time for the epidemic to reach its peak in larger nations (S3-S8 Tables in S1 File).

**Fig 3:**
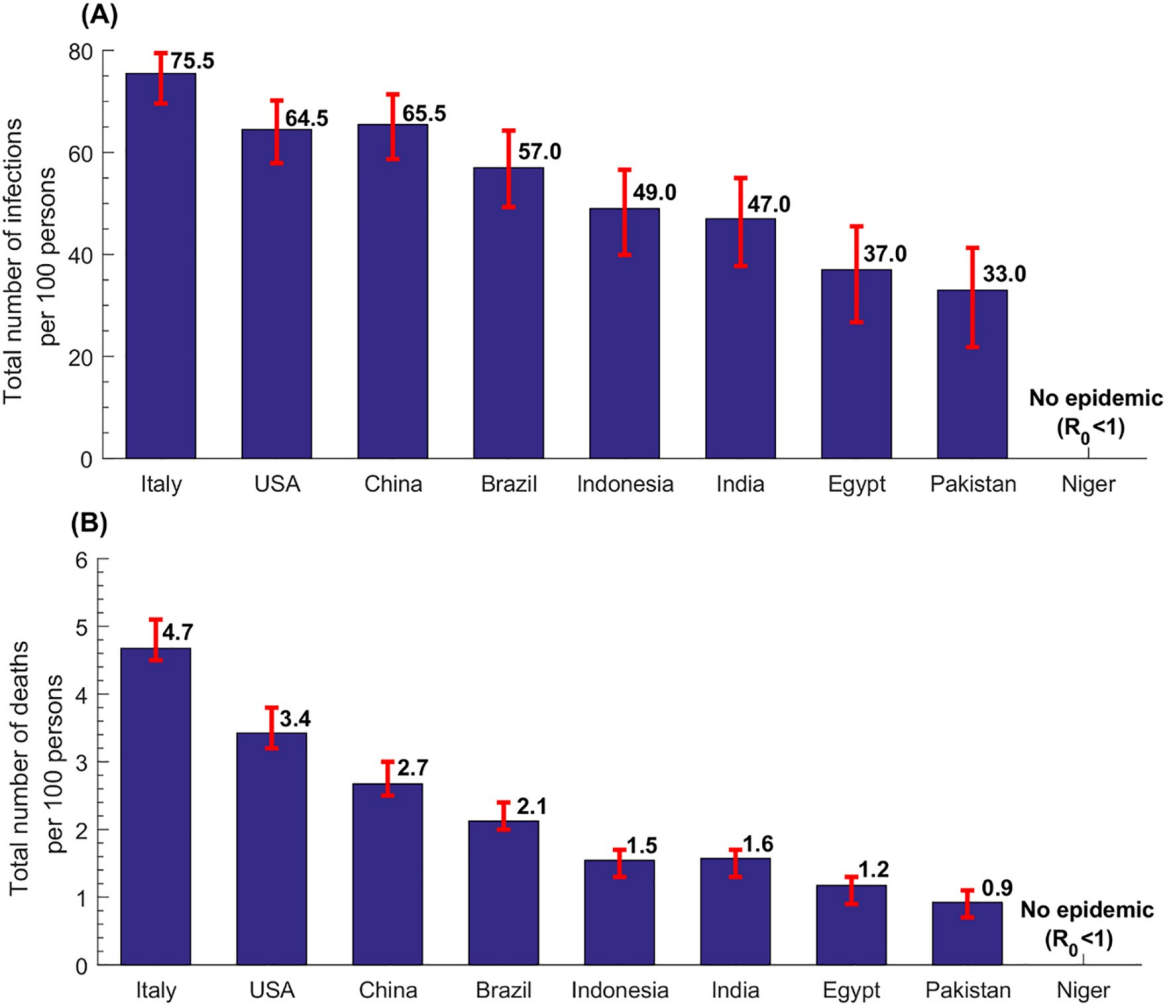
Estimates for the total number of infections and the total number of deaths per 100 persons in select countries.

The estimated incidence rate per 100 persons in the nine select countries, defined as the *cumulative* number of infections by the end of the epidemic cycle out of the total population, was highest in Italy at 75.5 and lowest in Pakistan at 33.0 (Fig 3A). Across world regions, the median incidence rate per 100 persons was 22.5 (range: 0.25–63.5) in AFRO, 45.0 (range: 1.5–65.0) in EMRO, 49.0 (range: 25.0–66.5) in SEARO, 53.0 in AMRO (range: 35.0–70.5), 63.5 (range: 31.0–76.5) in WPRO, and 69.0 (range: 31.0–75.5) in EURO, and 49.0 (range: 0.25–76.5) globally.

The estimated death rate per 100 persons in the nine select countries followed the same pattern, where it was highest in Italy at 4.7 and lowest in Pakistan at 0.9 (Fig 3B). Across world regions, the median death rate per 100 persons was 0.5 (range: 0.0–2.8) in AFRO, 0.9 (range: 0.0–2.0) in EMRO, 1.6 (range: 0.8–2.9) in SEARO, 2.0 in AMRO (range: 1.0–4.2), 2.7 (range: 0.8–5.3) in WPRO, and 3.9 (range: 0.8–4.7) in EURO, and 1.6 (range: 0.0–5.3) globally.

**Fig 4:**
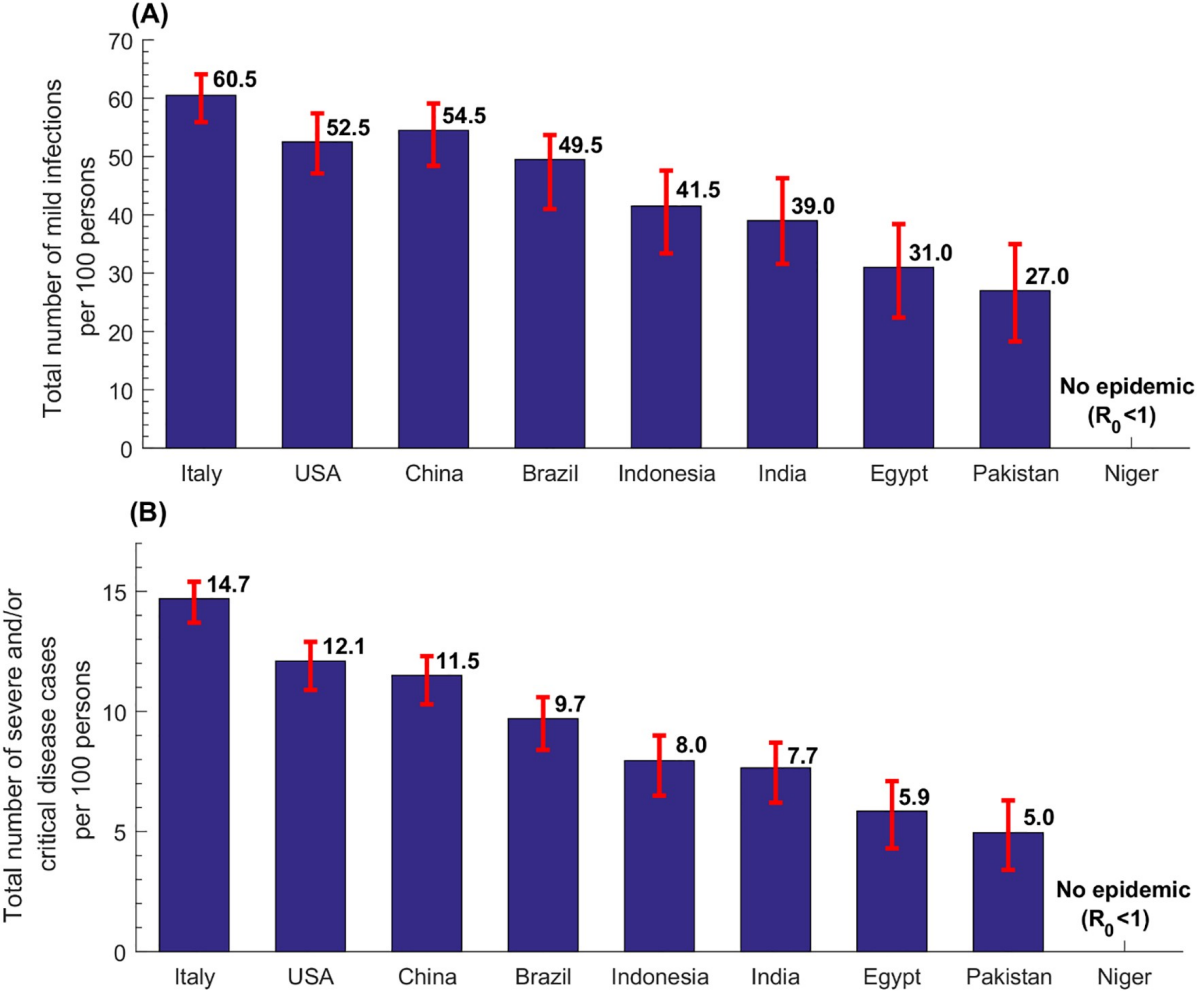
Estimates for the total number of mild infections and the total number of severe and/or critical diseases cases per 100 persons in select countries.

The estimated rate of mild infections per 100 persons in the nine select countries was highest in Italy at 60.5 and lowest in Pakistan at 27.0 (Fig 4A). Across world regions, the median per 100 persons was 17.0 (range: 0.25–52.5) in AFRO, 39.0 (range: 1.5–55.0) in EMRO, 41.0 (range: 23.0–54.5) in SEARO, 43.5 in AMRO (range: 29.0–56.5), 51.5 (range: 27.0–61.5) in WPRO, and 56.5 (range: 25.0–65.0) in EURO, and 41.0 (range: 0.25–61.5) globally.

Meanwhile, the estimated rate of severe and/or critical disease cases per 100 persons in the nine select countries was highest in Italy at 14.7 and lowest in Pakistan at 5.0 (Fig 4B). Across world regions, the median per 100 persons was 3.3 (range: 0.0–11.3) in AFRO, 7.1 (range: 0.3–9.5) in EMRO, 7.7 (range: 4.1–11.9) in SEARO, 8.7 in AMRO (range: 5.0–13.5), 11.5 (range: 5.0–15.5) in WPRO, and 13.0 (range: 4.7–14.7) in EURO, and 8.0 (range: 0.0–15.5) globally.

## Discussion

The above results suggest that although this pandemic is a formidable challenge globally, its intensity and toll in terms of morbidity and mortality may vary substantially by country and regionally. While some countries, particularly in resource-rich countries, may experience large and rapid epidemics, other countries, particularly in resource-poor countries, may experience smaller and slower epidemics. While the scale of the epidemic may be smallest in sub-Saharan Africa with its young population, it could be intense in countries with a relatively small proportion of children and sizable proportions of adults and/or elderly.

The key finding of this study is the potential role of age in driving divergent SARS-CoV-2 epidemic trajectories and disease burdens. The effect of age reflects the interplay of three main factors: the variation in susceptibility by age (S1 Fig in S1 File), variation in disease severity and mortality by age (S2 Fig in S1 File), and variation in demographic structure across countries (S3-S8 Tables in S1 File). The combined effect of these factors can result in stark variations in *R*_0_ (Fig 2), and consequently epidemic potential, disease severity, and disease mortality—there was strong correlation between *R*_0_ and median age across countries (S4 Fig in S1 File). Incidentally, the globally diagnosed cases as of July 16, 2020 show a significant correlation with median age across countries (Spearman correlation coefficient: 0.34 (95% CI: 0.19–0.47), p<0.001). Although this may be explained by more diagnosed infections being reported by countries with advanced testing infrastructure, this may support the role of age presented here. Importantly, as infection transmission dynamics is a non-linear phenomenon, even small changes in *R*_0_, when *R*_0_ is in the range of 1–2, can drive considerable differences in epidemic size and trajectory. Variability by age, though of different magnitude, was also noted in a recent other research work [7].

An illustration of the role of age can be seen in Niger, a nation with a median age of only 15 years, where the exclusion of children from the population, while maintaining the same susceptibility profile for the other age groups, would have increased *R*_0_ from 0.93 to 2.6. This demonstrates how the lower susceptibility among younger persons, particularly children, acts as a “herd immunity” impeding the ferocious strength of the force of infection of an otherwise very infectious virus. This epidemiological feature contrasts that of other respiratory infections, such as the 2009 influenza A (H1N1) pandemic (H1N1pdm) infection (S5 Fig in S1 File) [29], where the cumulative incidence was found to be highest among children and young adults, and much smaller among older adults.

One possible inference to be drawn from the above results is that epidemic size and associated disease burden could be highest in settings with sizable mid-age and/or elderly populations, as currently exemplified by Italy. This may also possibly explain sub-national patterns, such as the large number of diagnosed cases in the city of New York, although additional fine-grained analyses are needed to delineate within-country heterogeneities in transmission dynamics.

Another possible inference of relevance to containment efforts relates to the likelihood of the infection establishing itself in a population. In addition to the role of travel patterns/restrictions and multiple introductions for seeding the infection [30], the probability of a major outbreak upon introduction of one infection is given approximately by 1−1/*R*_0_ [31, 32]. Accordingly, in countries where *R*_0_ is just above the epidemic threshold of *R*_0_ = 1, the virus will need to be introduced multiple times before it can generate sustainable chains of transmission. In such countries, less disruptive social distancing measures may be sufficient to contain the epidemic compared to countries with larger *R*_0_.

Our study explores the potential effect of age on the epidemiology of SARS-CoV-2, but other factors that remain poorly understood, may also contribute to driving different epidemic trajectories. Transmission of the virus may be affected by seasonality, environmental and genetic factors, differences in social network structure and cultural norms (such as shaking hands, kissing, and other person-to-person contacts), age-dependent transmissibility effects, and underlying co-morbidities which also impact disease severity and mortality [33–36]. These factors may have contributed to a slowly growing epidemic in Japan, despite its demographic structure, as opposed to fast growing epidemics in the European Region and the USA.

This study has limitations. Model estimations are contingent on the validity and generalizability of input data. Our estimates were based on SARS-CoV-2 natural history and disease progression data from China, but these may not be applicable to other countries. The key factor driving the heterogeneity in transmission dynamics is the age-specific biological susceptibility profile. We used the susceptibility profile as derived from the China outbreak data [4], but this may not be generalizable to other countries. More estimates from other countries are needed to investigate the potential variation in this profile across countries, and different analytical approaches may also arrive at different estimates [7]. Some of the effects of biological susceptibility may simply reflect differences in social behavior or the probability of infection ascertainment. The presence and contribution of such factors are, however, difficult to disentangle.

Model 10-year age-bands were informed by available empirical evidence, which hindered analysis using narrower age intervals [37]. While current data on the attack rate from different countries supports age heterogeneity and lower exposure among children, different countries show still variation in the age-stratified attack rate (S6, S7 Figs in S1 File). Of note that the observed lower susceptibility to the infection at younger age [4] does not necessarily imply absence of infection, but may reflect rapid infection clearance or subclinical infection. This may impact our estimates depending on these infections’ degree of infectiousness. Two sensitivity analyses assuming a 50% increase in the susceptibility of younger age cohorts or equal susceptibility among those aged <20 to those aged 20–29 (to somewhat reflect the effect of subclinical infections) resulted in smaller differences in *R*_0_ across countries, but still supported our findings of wide heterogeneity in *R*_0_ (S8 and S9 Figs in S1 File, respectively). It is conceivable that such subclinical infections may have lower viral load, and therefore rapid infection clearance and limited transmission potential. Data on infection clusters from China and other countries suggested that children do not appear to play a significant role in the transmission of this infection [7, 8, 38].

Increasingly, evidence suggests that *R*_0_ is higher than assumed here based on fitting the China epidemic [4, 39, 40], which would increase the *R*_0_ values reported here, but will not affect the relative differences across countries. We assumed a constant overall contact rate across countries, but contact rate patterns, and therefore *R*_0_, can vary across and within countries [34, 35], and also over time with the implementation of interventions and associated behavioral changes [41]. There could be also transient effects affecting transmission patterns and epidemic dynamics [41, 42]. We assumed low degree of assortativeness in age group mixing based on fitting the China epidemic [4], however, a sensitivity analysis assuming high assortativeness, though yielded higher estimates for *R*_0_, still preserved the relative differences in these estimates across countries (S10 Fig in S1 File).

Our study may have overestimated disease mortality by basing mortality rates on estimates of the crude case fatality rate in China, as suggested by a recent study [13]. Our results are based on the most probable value for *R*_0_ as estimated through 500 runs of uncertainty analysis. The latter may have biased our reported results towards lower *R*_0_. For instance, the most probable value for *R*_0_ in China was 1.95, but the point estimate assuming the baseline values of the input parameters was higher at 2.10 [4]. Despite these limitations, our parsimonious model, tailored to the nature of available data, was able to reproduce the epidemic as observed in China [4], and generated results that are valid to a wide range of model assumptions.

Age appears to be a driver of variable SARS-CoV-2 epidemic trajectories worldwide. Countries with sizable adult and/or elderly populations and smaller children populations may experience large and rapid epidemics in absence of interventions, necessitating disruptive social distancing measures to contain the epidemic. Meanwhile, countries with predominantly younger age cohorts may experience smaller and slower epidemics and may require less disruptive social distancing measures to contain the epidemic. These predictions, however, should not lead to complacency, and should not affect national response in strengthening preparedness plans and in implementing prevention interventions, as although many countries are predicted to have lower *R*_0_, these also often coincide with those having limited healthcare capacity and testing. Once established, and even if smaller in scale due to their predominantly younger population, the SARS-CoV-2 epidemic would still cause a heavy toll on developing countries with resource-poor healthcare infrastructure.

## Supporting information

S1 File (DOCX)
