# Supplementary material for: Age could be driving variable SARS-CoV-2 epidemic trajectories worldwide
Source: PLoS One. 2020 Aug 20;15(8):e0237959. doi: 10.1371/journal.pone.0237959 (PMC7444586; doi:10.1371/journal.pone.0237959)
Supplement: S1 File — (DOCX) [file pone.0237959.s001.docx]

Age could be driving variable SARS-CoV-2 epidemic trajectories worldwide

Table of Contents

**S1 Fig.** Age-specific susceptibility to SARS-CoV-2 acquisition relative to individuals aged 60-69 years 3

**S2 Fig.** Key attributes of SARS-CoV-2 disease progression stratified by age 4

**Section 1.** Mathematical model structure 5

**S3 Fig.** Schematic diagram illustrating the basic structure of the SARS-CoV-2 model 5

**S1 Table.** Definitions of population variables and symbols used in the model 7

**Section 2.** Parameter values 8

**S2 Table.** Model assumptions in terms of parameter values 8

**Section 3.** The basic reproduction number *R*_0_ 10

**S3 Table.** Model estimates for key SARS-CoV-2 epidemiologic indicators for countries and territories with a population of at least one million in the World Health Organization African Region 11

**S4 Table.** Model estimates for key SARS-CoV-2 epidemiologic indicators for countries and territories with a population of at least one million in the World Health Organization Region of the Americas 13

**S5 Table.** Model estimates for key SARS-CoV-2 epidemiologic indicators for countries and territories with a population of at least one million in the World Health Organization Eastern Mediterranean Region 14

**S6 Table.** Model estimates for key SARS-CoV-2 epidemiologic indicators for countries and territories with a population of at least one million in the World Health Organization European Region 15

**S7 Table.** Model estimates for key SARS-CoV-2 epidemiologic indicators for countries and territories with a population of at least one million in the World Health Organization South-East Asia Region 17

**S8 Table.** Model estimates for key SARS-CoV-2 epidemiologic indicators for countries and territories with a population of at least one million in the World Health Organization Western Pacific Region 18

**S4 Fig.** Impact of the variation in the median age on the basic reproduction number *R*_0_ 19

**S5 Fig.** Age-specific cumulative incidence of the 2009 influenza A (H1N1) pandemic (H1N1pdm) virus 20

**S6 Fig.** Distribution of SARS-CoV-2 age-specific attack rate per 10,000 persons for Australia, Austria, Denmark, Finland, France, Germany, Iceland, and Italy 21

**S7 Fig.** Distribution of SARS-CoV-2 age-specific attack rate per 10,000 persons for Japan, Norway, Singapore, Sweden, and Republic of Korea 22

**S8 Fig.** Sensitivity analysis assessing the impact of a 50% increase in the susceptibility to SARS-CoV-2 infection among those aged <30 years on our estimates for the basic reproduction number, *R_0_*, for the select countries presented in the main text 23

**S9 Fig.** Sensitivity analysis assessing the impact of equal susceptibility to SARS-CoV-2 infection among those aged <20 to those aged 20-29 years on our estimates for the basic reproduction number, *R_0_*, for the select countries presented in the main text 24

**S10 Fig.** Sensitivity analysis assessing the impact of high degree of assortativeness in the age group mixing on our estimates for the basic reproduction number, *R_0_*, for the select countries presented in the main text 25

**References** 26

**S1 Fig. Age-specific susceptibility to SARS-CoV-2 acquisition relative to individuals aged 60-69 years.** This figure was reproduced from *Ayoub et al.* [1], with susceptibility being normalized relative to those aged 60-69 years.


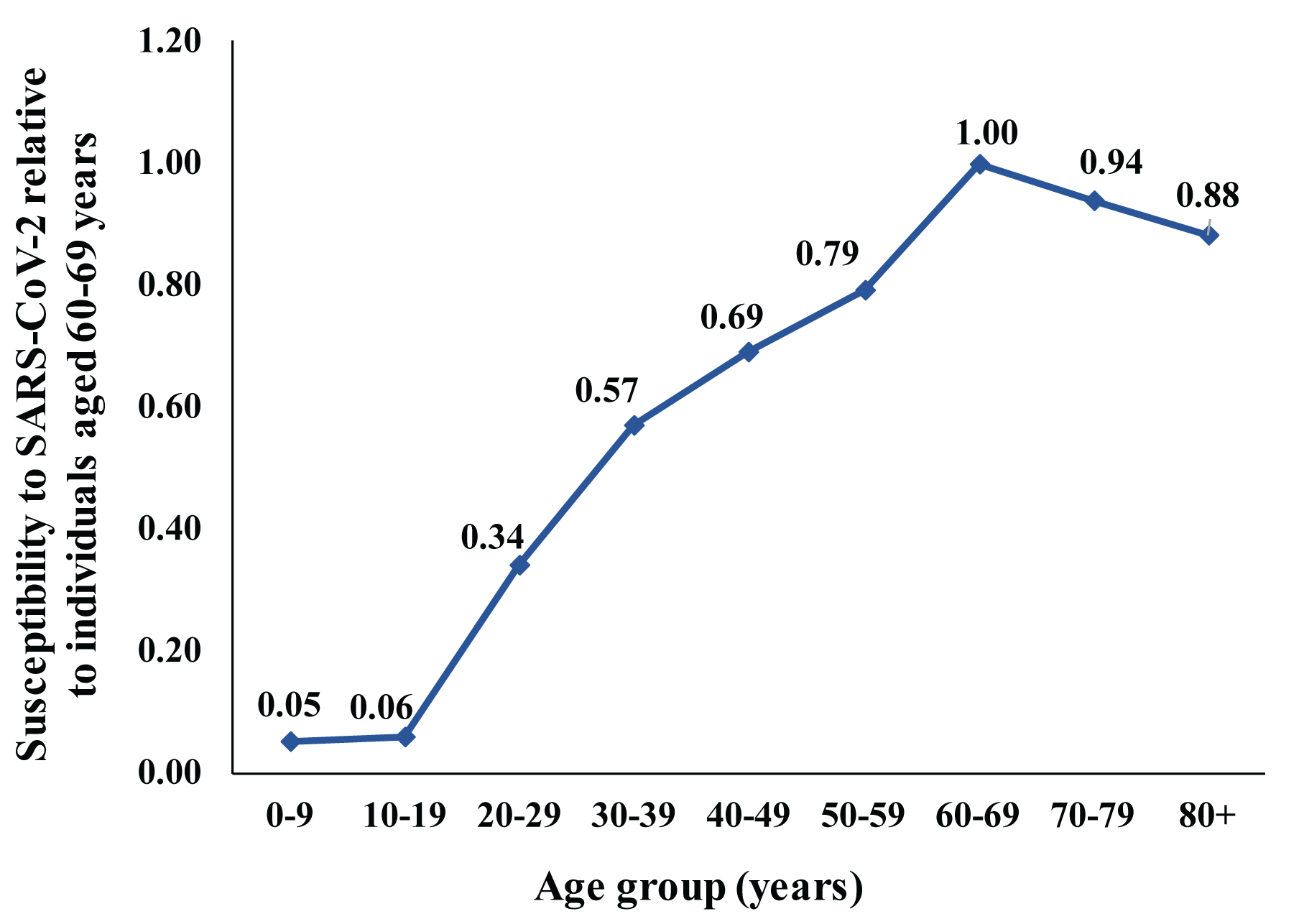


**Fig S2. Key attributes of SARS-CoV-2 disease progression stratified by age.** Distribution by age of: A) the proportion of infections that will progress to be mild or asymptomatic, severe, or critical [2-4] and B) crude case fatality rate [5, 6]. This figure was reproduced from results of references [2-6].


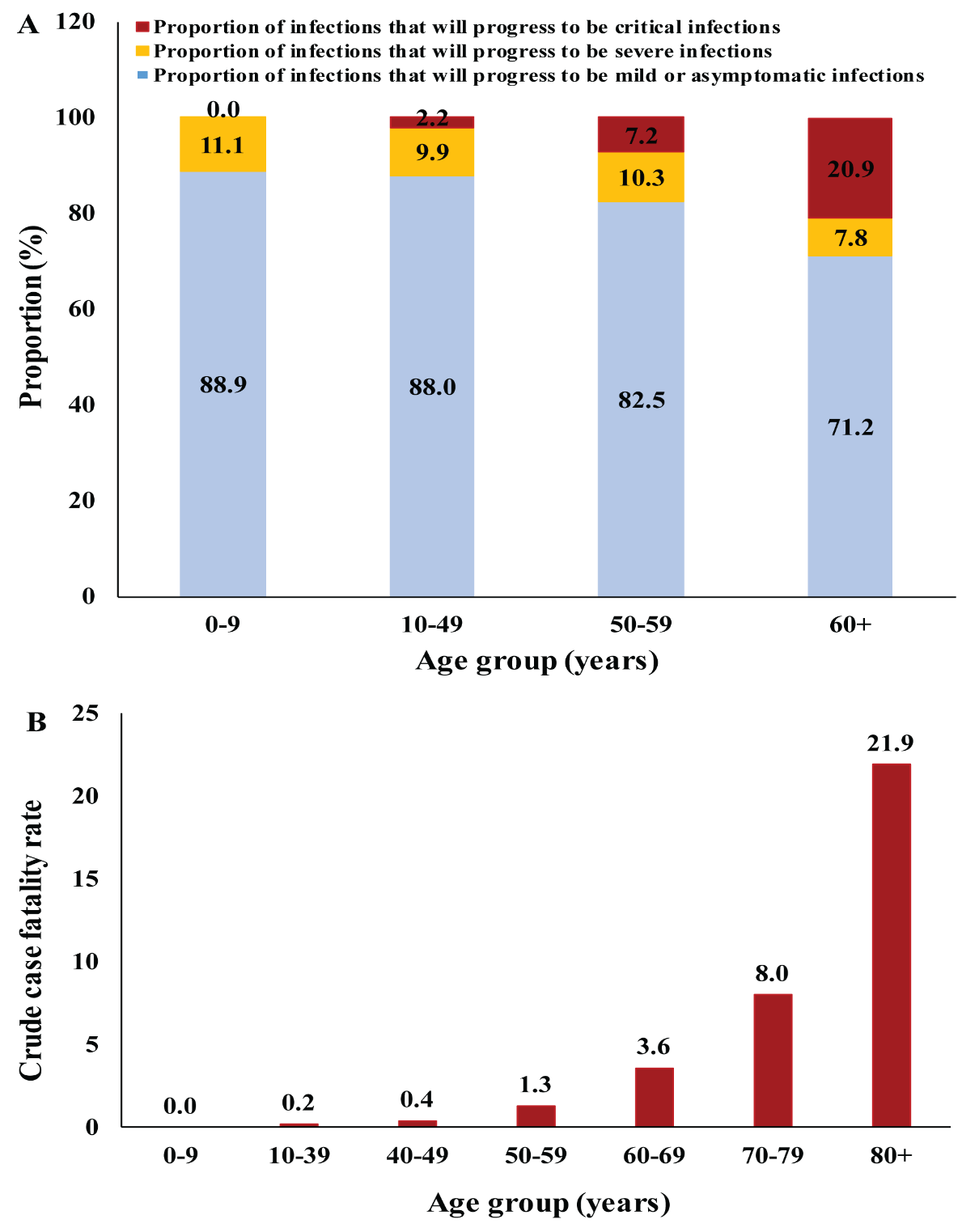


1. **Mathematical model structure**

We applied a recently developed deterministic compartmental mathematical model that describes the [severe acute respiratory syndrome coronavirus 2](https://en.wikipedia.org/wiki/Severe_acute_respiratory_syndrome_coronavirus_2) (SARS-CoV-2) transmission dynamics and disease progression in a population [1], to all countries and territories with a population of at least one million, as of 2020 [7]. An illustration of the basic model structure can be found in S3 Fig. The model stratifies the population into compartments based on age (0-9, 10-19, 20-29,…, ≥80 years), infection status (uninfected, infected), infection stage (mild, severe, critical), and disease stage (severe, critical).

**S3 Fig. Schematic diagram illustrating the basic structure of the SARS-CoV-2 model.**

**
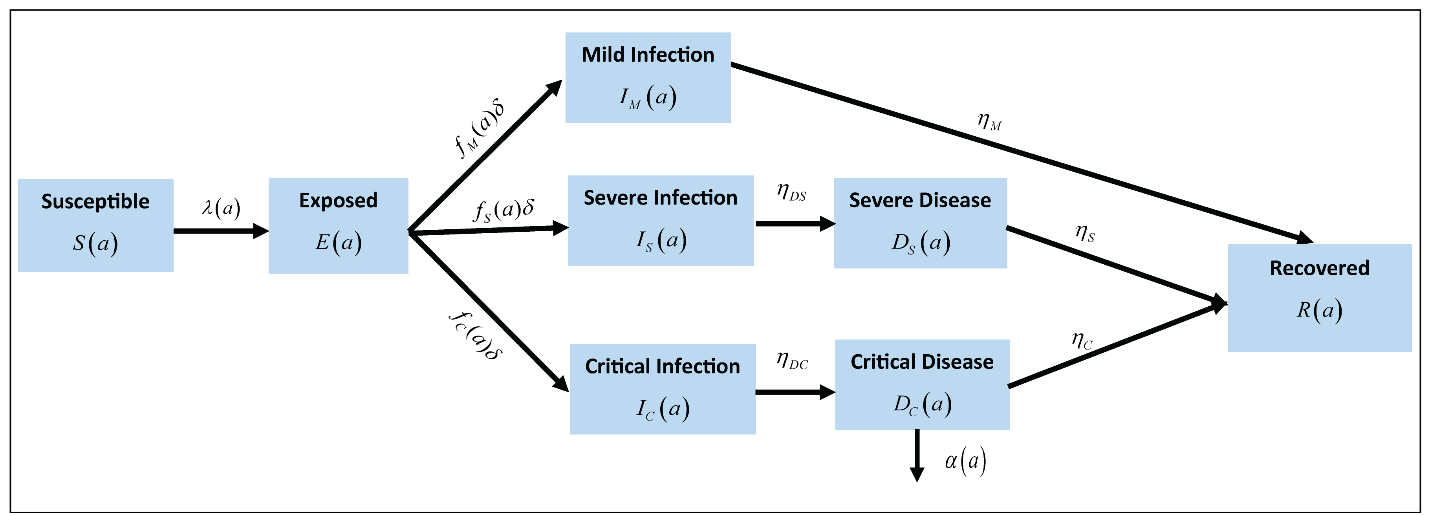
**

A system of coupled nonlinear differential equations is used to describe SARS-CoV-2 transmission dynamics. Nine age cohorts were considered $\left(a=1,2,...,9\right)$, each representing a ten-year age band apart from the last cohort which includes individuals aged ≥80 years. The age-specific distribution for each country/territory, as of the year 2020, was obtained from the United Nations World Population Prospects database [7]. All disease-related mortality was assumed to occur in individuals that are in the critical disease stage, as informed by the China outbreak data [5].

The epidemic dynamics in the first age group was described using:

Population aged 0-9 years:

$$\frac{dS(1)}{dt}=-\left(\lambda\left(1\right)+\mu+\xi\left(1\right)\right)S(1)$$

$$\frac{dE(1)}{dt}=\lambda\left(1\right)S\left(1\right)-\left(\delta+\mu+\xi\left(1\right)\right)E(1)$$

$$\frac{dI_{M}(1)}{dt}=f_{M}(1)\delta E\left(1\right)-\left(\eta_{M}+\mu+\xi\left(1\right)\right)I_{M}(1)$$

$$\frac{dI_{S}(1)}{dt}=f_{S}(1)\delta E\left(1\right)-\left(\eta_{DS}+\mu+\xi\left(1\right)\right)I_{S}(1)$$

$$\frac{dI_{C}(1)}{dt}=f_{C}(1)\delta E\left(1\right)-\left(\eta_{DC}+\mu+\xi\left(1\right)\right)I_{C}(1)$$

$$\frac{dD_{S}(1)}{dt}=\eta_{DS}I_{S}\left(1\right)-\left(\eta_{S}+\mu+\xi\left(1\right)\right)D_{S}(1)$$

$$\frac{dD_{C}(1)}{dt}=\eta_{DC}I_{C}\left(1\right)-\left(\eta_{C}+\mu+\xi\left(1\right)+\alpha\left(1\right)\right)D_{C}(1)$$

$$\frac{dR(1)}{dt}=\eta_{M}I_{M}(1)+\eta_{S}D_{S}(1)+\eta_{C}D_{C}(1)-\left(\mu+\xi\left(1\right)\right)R(1)$$

and in subsequent age groups, using:

Populations aged 10+ years:

$$\frac{dS(a)}{dt}=\xi\left(a-1\right)S(a-1)-\left(\lambda\left(a\right)+\mu+\xi\left(a\right)\right)S(a)$$

$$\frac{dE(a)}{dt}=\xi\left(a-1\right)E(a-1)+\lambda\left(a\right)S\left(a\right)-\left(\delta+\mu+\xi\left(a\right)\right)E(a)$$

$$\frac{dI_{M}(a)}{dt}=\xi\left(a-1\right)I_{M}(a-1)+f_{M}(a)\delta E\left(a\right)-\left(\eta_{M}+\mu+\xi\left(a\right)\right)I_{M}(a)$$

$$\frac{dI_{S}(a)}{dt}=\xi\left(a-1\right)I_{S}(a-1)+f_{S}(a)\delta E\left(a\right)-\left(\eta_{DS}+\mu+\xi\left(a\right)\right)I_{S}(a)$$

$$\frac{dI_{C}(a)}{dt}=\xi\left(a-1\right)I_{C}(a-1)+f_{C}(a)\delta E\left(a\right)-\left(\eta_{DC}+\mu+\xi\left(a\right)\right)I_{C}(a)$$

$$\frac{dD_{S}(a)}{dt}=\xi\left(a-1\right)D_{S}(a-1)+\eta_{DS}I_{S}\left(a\right)-\left(\eta_{S}+\mu+\xi\left(a\right)\right)D_{S}(a)$$

$$\frac{dD_{C}(a)}{dt}=\xi\left(a-1\right)D_{C}(a-1)+\eta_{DC}I_{C}\left(a\right)-\left(\eta_{C}+\mu+\xi\left(a\right)+\alpha\left(a\right)\right)D_{C}(a)$$

$$\frac{dR(a)}{dt}=\xi\left(a-1\right)R(a-1)+\eta_{M}I_{M}(a)+\eta_{S}D_{S}(a)+\eta_{C}D_{C}(a)-\left(\mu+\xi\left(a\right)\right)R(a)$$

The definitions of the population variables and symbols used in the equations are listed in S1Table.

**S1 Table.** **Definitions of population variables and symbols used in the model.**

| **Symbol** | **Definition** |
| --- | --- |
| $S(a)$ | Susceptible population |
| $E(a)$ | Latently infected population |
| $I_{M}\left(a\right)$ | Population with mild infection |
| $I_{S}\left(a\right)$ | Population with severe infection |
| $I_{C}\left(a\right)$ | Population with critical infection |
| $D_{S}\left(a\right)$ | Population with severe disease |
| $D_{C}\left(a\right)$ | Population with critical disease |
| $R(a)$ | Recovered population |
| $N$ | Total population size |
| $n_{age}$ | Number of age groups |
| $\xi(a)$ | Transition rate from one age group to the next age group |
| $\sigma\left(a\right)$ | Susceptibility profile to the infection in each age group |
| $\beta\left(t\right)$ | Average infectious contact rate |
| $e_{Age}$ | Degree of assortativeness in the age group mixing |
| $1/\delta$ | Duration of latent infection |
| $1/\eta_{M}$ | Duration of mild infection infectiousness |
| $1/\eta_{DS}$ | Duration of severe infection infectiousness before isolation and/or hospitalization |
| $1/\eta_{S}$ | Duration of severe disease following onset of severe disease |
| $1/\eta_{DC}$ | Duration of critical infection infectiousness before isolation and/or hospitalization |
| $1/\eta_{C}$ | Duration of critical disease following onset of critical disease |
| $1/\mu$ | Natural death rate |
| $CFR\left(a\right)$ | *Relative* case fatality rate in each age group |
| $\alpha\left(a\right)$ | Mortality rate in each age group |
| $f_{M}(a)$ | Proportion of infections that will progress to be mild or asymptomatic infections |
| $f_{S}(a)$ | Proportion of infections that will progress to be severe infections |
| $f_{C}(a)$ | Proportion of infections that will progress to be critical infections |

The force of infection (hazard rate) for each susceptible population in each age group $S(a)$ is given by

$\lambda\left(a\right)=\beta\left(t\right)\sigma\left(a\right)\sum_{a'=1}^{n_{age}} H_{a,a'}\frac{I_{M}(a')+I_{S}(a')+I_{C}(a')}{\left[S(a')+E(a')+I_{M}(a')+I_{S}(a')+I_{C}(a')+D_{S}(a')+D_{C}(a')+R(a')\right]}$,

where $\beta\left(t\right)$ is the overall infectious contact rate per day, $\sigma\left(a\right)$ is the susceptibility profile to the infection in each age group, and $H_{a,a'}$ is the mixing matrix which provides the probability that an individual in the $a$ age group will mix with (that is contact) an individual in the $a'$ age group. The mixing matrix is given by

$$H_{a,a'}=e_{Age}\delta_{a,a'}+(1-e_{Age})\frac{S(a')+E(a')+I_{M}(a')+I_{S}(a')+I_{C}(a')+D_{S}(a')+D_{C}(a')+R(a')}{\sum_{a''=1}^{n_{age}} \left[S(a'')+E(a'')+I_{M}(a'')+I_{S}(a'')+I_{C}(a'')+D_{S}(a'')+D_{C}(a'')+R(a'')\right]}$$

Here, $\delta_{a,a'}$ is the identity matrix, and $e_{Age}\in\left[0,1\right]$ is the degree of assortativeness in the mixing. At the extreme $e_{Age}=0$, the mixing is fully proportional, while at the other extreme $e_{Age}=1$, the mixing is fully assortative, that is individuals mix only with members in their own age group.

1. **Parameter values**

The input parameters of the model were chosen based on current empirical data for SARS-CoV-2 natural history and epidemiology. The parameter values are listed in S2 Table.

**S2 Table.** **Model assumptions in terms of parameter values.**

| **Parameter** | **Symbol** | **Value** | **Justification** |
| --- | --- | --- | --- |
| Duration of latent infection | $1/\delta$ | 3.69 days | Based on existing estimate [8]. This estimate is consistent with observed median incubation period of 5.1 days [9] and distribution of viral load versus time since onset of infection [10, 11] |
| Duration of infectiousness | $1/\eta_{M}$;$1/\eta_{DS}$; $1/\eta_{DC}$ | 3.48 days | Based on existing estimate [8]. This estimate is consistent with observed time to recovery among persons with mild infection [4, 8] and distribution of viral load versus time since onset of infection [10, 11] |
| Duration of severe disease following onset of severe disease | $1/\eta_{S}$ | 28 days | Observed duration from onset of severe disease to recovery [4] |
| Duration of hospitalization for critical infection | $1/\eta_{C}$ | 28 days | Observed duration from onset of critical disease to recovery [4] |
| Life expectancy for each country | $1/\mu$ | S3 Table | United Nations World Population Prospects database [7] |
| *Relative* case fatality rate in each age group | $CFR\left(a\right)$ |  | Observed crude case fatality rate based on China data [5, 6] |
| Age 0-9 years |  | 0% | Supported by empirical evidence [12-14] |
| Age 10-39 years |  | 0.2% |  |
| Age 40-49 years |  | 0.4% |  |
| Age 50-59 years |  | 1.3% |  |
| Age 60-69 years |  | 3.6% |  |
| Age 70-79 years |  | 8.0% |  |
| Age 80+ years |  | 21.9% |  |
| Proportion of infections that will progress to be mild or asymptomatic infections | $f_{M}(a)$ |  | Observed proportion of infections that eventually develop mild or asymptomatic in China [2-4] |
| Age 0-9 years |  | 88.9% |  |
| Age 10-49 years |  | 88.0% |  |
| Age 50-59 years |  | 82.5% |  |
| Age 60+ years |  | 71.2% |  |
| Proportion of infections that will progress to be severe infections | $f_{S}(a)$ |  | Observed proportion of infections that eventually develop severe disease in China [2-4] |
| Age 0-9 years |  | 11.1% |  |
| Age 10-49 years |  | 9.9% |  |
| Age 50-59 years |  | 10.3% |  |
| Age 60+ years |  | 7.8% |  |
| Proportion of infections that will progress to be critical infections | $f_{C}(a)$ |  | Observed proportion of infections that eventually develop critical disease in China [2-4] |
| Age 0-9 years |  | 0.0% |  |
| Age 10-49 years |  | 2.2% |  |
| Age 50-59 years |  | 7.2% |  |
| Age 60+ years |  | 20.9% |  |
| Susceptibility profile to the infection in each age group | $\sigma\left(a\right)$ |  | Estimates based on fitting the epidemic in China [1]. Values are as estimated by the model and not with reference to any specific age group. |
| Age 0-9 years |  | 0.05 |  |
| Age 10-19 years |  | 0.05 |  |
| Age 20-29 years |  | 0.32 |  |
| Age 30-39 years |  | 0.53 |  |
| Age 40-49 years |  | 0.65 |  |
| Age 50-59 years |  | 0.74 |  |
| Age 60-69 years |  | 0.93 |  |
| Age 70-79 years |  | 0.88 |  |
| Age 80+ years |  | 0.83 |  |
| Overall infectious contact rate | $\beta\left(t\right)$ | 0.59 contacts per day | Estimate based on fitting the epidemic in China [1] |
| Degree of assortativeness in the age group mixing | $e_{Age}$ | 0.004 | Estimate based on fitting the epidemic in China [1] |

1. **The basic reproduction number *R_0_***

Using the second generation matrix method described by Heffernan *et al.* [15], the basic reproduction number in the special case of proportional mixing (no assortativeness) was derived to be:

$R_{0}=\sum_{a=1}^{n_{age}} \left[\begin{matrix} w(a)f_{M}(a)\left(\frac{\delta}{\delta+\mu+\xi\left(a\right)}\frac{\beta\sigma\left(a\right)}{\eta_{M}+\mu+\xi\left(a\right)}\right) \\ +w\left(a\right)f_{S}(a)\left(\frac{\delta}{\delta+\mu+\xi\left(a\right)}\frac{\beta\sigma\left(a\right)}{\eta_{DS}+\mu+\xi\left(a\right)}\right) \\ +w\left(a\right)f_{C}(a)\left(\frac{\delta}{\delta+\mu+\xi\left(a\right)}\frac{\beta\sigma\left(a\right)}{\eta_{DC}+\mu+\xi\left(a\right)}\right) \end{matrix}\right]$,

where $w(a)$ is the proportion of the population in each age group.

**S3 Table. Model estimates for key SARS-CoV-2 epidemiologic indicators for countries and territories with a population of at least one million [7] in the World Health Organization African Region (AFRO).**

| African Region | Total population [7] | Med-ian age [7] | Life expec-tancy [7] | *R*_0_^a^ | Infections per 100 persons^a^ | Deaths per 100 persons^a^ | Mild infections per 100 persons^a^ | Severe and/or critical disease cases per 100 persons^a^ | Day at peak incidence^a^ |
| --- | --- | --- | --- | --- | --- | --- | --- | --- | --- |
| Country | N | Years | Years | N (95% UI) | N (95% UI) | N (95% UI) | N (95% UI) | N (95% UI) | N (95% UI) |
| Algeria | 43,851,043 | 28.5 | 77.5 | 1.45 (1.35-2.21) | 45.0 (35.2-52.6) | 1.4 (1.2-1.6) | 37.0 (29.5-44.3) | 7.4 (5.7-8.3) | 217.5 (206.0-246.0) |
| Angola | 32,866,268 | 16.7 | 62.2 | 0.98 (0.88-1.46) | 1.5 (0.0-23.4) | 0.0 (0.0-0.5) | 1.0 (0.0-20.0) | 0.2 (0.0-3.4) | 697.5 (518.0-700^b^) |
| Benin | 12,123,198 | 18.8 | 62.8 | 1.05 (0.98-1.61) | 19.5 (0.1-29.8) | 0.6 (0.0-0.7) | 17.0 (0.1-25.4) | 3.3 (0.0-4.5) | 675.0 (360.0-700^b^) |
| [Botswana](http://www.who.int/countries/bwa/en/) | 2,351,625 | 24.0 | 69.9 | 1.25 (1.18-1.94) | 35.0 (23.6-43.6) | 0.9 (0.7-1.1) | 29.0 (19.9-37.0) | 5.3 (3.7-6.6) | 235.0 (212.0-306.0) |
| [Burkina Faso](http://www.who.int/countries/bfa/en/) | 20,903,278 | 17.6 | 63.0 | 0.98 (0.91-1.51) | 2.5 (0.0-25.6) | 0.1 (0.0-0.5) | 1.5 (0.0-21.9) | 0.3 (0.0-3.8) | 700^b^ (453.0-700^b^) |
| Burundi | 11,890,781 | 17.3 | 62.7 | 1.03 (0.90-1.49) | 2.5 (0.0-24.8) | 0.1 (0.0-0.5) | 1.5 (0.0-21.1) | 0.3 (0.0-3.6) | 700^b^ (454.0-700^b^) |
| Cameroon | 26,545,864 | 18.7 | 60.3 | 1.05 (0.96-1.59) | 2.5 (0.0-29.2) | 0.1 (0.0-0.6) | 17.5 (0.0-24.9) | 0.3 (0.0-4.3) | 675.0 (397.0-700^b^) |
| Cent. Afr. Rep. | 4,829,764 | 17.6 | 54.4 | 0.98 (0.91-1.50) | 2.5 (0.0-24.7) | 0.1 (0.0-0.6) | 1.5 (0.0-21.0) | 0.3 (0.0-3.7) | 700^b^ (420.0-700^b^) |
| Chad | 16,425,859 | 16.6 | 55.2 | 0.92 (0.86-1.43) | 1.0 (0.0-22.0) | 0.0 (0.0-0.5) | 1.0 (0.0-18.7) | 0.2 (0.0-3.2) | 697.5 (532.0-700^b^) |
| Congo | 5,518,092 | 19.2 | 65.2 | 1.15 (1.01-1.66) | 23.0 (2.5-31.7) | 0.5 (0.1-0.7) | 19.0 (2.2-27.0) | 3.4 (0.4-4.7) | 405.0 (316.0-700^b^) |
| [Côte d'Ivoire](http://www.who.int/countries/civ/en/) | 26,378,275 | 18.9 | 58.8 | 1.05 (0.97-1.60) | 22.5 (0.0-29.6) | 0.6 (0.0-0.7) | 1.5 (0.0-25.2) | 3.3 (0.0-4.4) | 675.0 (389.0-700^b^) |
| [D. Rep. Congo](http://www.who.int/countries/cod/en/) | 89,561,404 | 17.0 | 61.6 | 0.98 (0.91-1.51) | 2.5 (0.0-25.4) | 0.1 (0.0-0.6) | 1.5 (0.0-21.6) | 0.3 (0.0-3.8) | 690.0 (497.0-700^b^) |
| [Equ. Guinea](http://www.who.int/countries/gnq/en/) | 1,402,985 | 22.3 | 59.8 | 1.15 (1.05-1.74) | 27.0 (14.7-36.7) | 0.5 (0.3-0.6) | 23.0 (12.6-31.6) | 3.8 (2.1-5.2) | 310.0 (259.0-534.0) |
| [Eritrea](http://www.who.int/countries/eri/en/) | 3,546,427 | 19.2 | 67.5 | 1.15 (1.01-1.67) | 23.0 (6.1-31.7) | 0.7 (0.2-0.9) | 19.0 (5.2-26.9) | 3.5 (1.0-4.9) | 345.0 (303.0-700^b^) |
| [Ethiopia](http://www.who.int/countries/eth/en/) | 114,963,583 | 19.5 | 67.8 | 1.05 (0.99-1.63) | 22.5 (0.0-31.5) | 0.7 (0.0-0.8) | 19.5 (0.0-26.8) | 3.8 (0.0-4.7) | 675.0 (402.0-700^b^) |
| Eswatini | 1,160,164 | 20.7 | 61.1 | 1.15 (1.04-1.72) | 25.0 (13.7-34.5) | 0.7 (0.4-0.8) | 23.0 (11.6-29.4) | 3.8 (2.1-5.1) | 330.0 (261.0-547.0) |
| [Gabon](http://www.who.int/countries/gab/en/) | 2,225,728 | 22.5 | 67.0 | 1.25 (1.11-1.83) | 29.0 (18.4-39.1) | 0.7 (0.5-0.9) | 25.0 (15.6-33.3) | 4.4 (2.8-5.8) | 275.0 (239.0-404.0) |
| [Gambia](http://www.who.int/countries/gmb/en/) | 2,416,664 | 17.8 | 63.3 | 1.03 (0.92-1.52) | 2.5 (0.0-25.5) | 0.1 (0.0-0.5) | 1.5 (0.0-21.8) | 0.3 (0.0-3.7) | 675.0 (383.0-700^b^) |
| Ghana | 31,072,945 | 21.5 | 64.9 | 1.15 (1.09-1.79) | 29.0 (17.7-37.7) | 0.7 (0.5-0.8) | 25.0 (15.0-32.1) | 4.4 (2.7-5.6) | 350.0 (298.0-538.0) |
| Guinea | 13,132,792 | 18.0 | 62.6 | 1.03 (0.93-1.53) | 2.5 (0.0-26.4) | 0.1 (0.0-0.6) | 2.5 (0.0-22.4) | 0.3 (0.0-3.9) | 700^b^ (423.0-700^b^) |
| Guinea-Bissau | 1,967,998 | 18.8 | 59.4 | 1.05 (0.97-1.60) | 19.0 (0.1-29.0) | 0.5 (0.0-0.6) | 17.0 (0.1-24.7) | 2.9 (0.0-4.3) | 675.0 (326.0-700^b^) |
| Kenya | 53,771,300 | 20.1 | 67.5 | 1.15 (1.01-1.67) | 25.0 (0.6-33.5) | 0.5 (0.0-0.6) | 21.0 (0.5-28.6) | 3.4 (0.1-4.9) | 425.0 (361.0-700^b^) |
| Lesotho | 2,142,252 | 24.0 | 55.7 | 1.25 (1.18-1.95) | 37.0 (23.9-43.9) | 1.0 (0.7-1.2) | 29.0 (20.2-37.2) | 5.6 (3.8-6.7) | 235.0 (210.0-299.0) |
| [Liberia](http://www.who.int/countries/lbr/en/) | 5,057,677 | 19.4 | 65.0 | 1.15 (1.01-1.67) | 23.0 (4.0-31.9) | 0.6 (0.1-0.7) | 21.0 (3.4-27.1) | 3.4 (0.6-4.8) | 375.0 (312.0-700^b^) |
| Madagascar | 27,691,019 | 19.6 | 68.2 | 1.05 (1.00-1.66) | 23.0 (0.6-32.1) | 0.6 (0.0-0.7) | 19.0 (0.5-27.3) | 3.4 (0.1-4.8) | 425.0 (355.0-700^b^) |
| Malawi | 19,129,955 | 18.1 | 65.6 | 1.03 (0.92-1.53) | 2.5 (0.0-26.7) | 0.1 (0.0-0.6) | 2.5 (0.0-22.8) | 0.3 (0.0-3.9) | 700^b^ (434.0-700^b^) |
| Mali | 20,250,834 | 16.3 | 60.5 | 0.98 (0.87-1.44) | 1.0 (0.0-22.3) | 0.0 (0.0-0.5) | 1.0 (0.0-19.0) | 0.2 (0.0-3.3) | 697.5 (526.0-700^b^) |
| [Mauritania](http://www.who.int/countries/mrt/en/) | 4,649,660 | 20.1 | 65.6 | 1.15 (1.03-1.70) | 25.0 (11.1-33.6) | 0.6 (0.3-0.8) | 21.0 (9.4-28.7) | 3.8 (1.7-5.0) | 375.0 (294.0-642.0) |
| [Mauritius](http://www.who.int/countries/mus/en/) | 1,271,767 | 37.5 | 75.5 | 1.95 (1.72-2.80) | 63.5 (56.7-69.9) | 2.8 (2.6-3.1) | 52.5 (46.6-57.7) | 11.3 (10.1-12.2) | 116.0 (115.0-117.0) |
| [Mozambique](http://www.who.int/countries/moz/en/) | 31,255,435 | 17.6 | 62.1 | 1.03 (0.92-1.51) | 2.5 (0.0-25.8) | 0.1 (0.0-0.6) | 1.5 (0.0-22.0) | 0.3 (0.0-3.8) | 700^b^ (462.0-700^b^) |
| [Namibia](http://www.who.int/countries/nam/en/) | 2,540,916 | 21.8 | 64.9 | 1.15 (1.08-1.79) | 31.0 (17.0-37.6) | 0.7 (0.5-0.9) | 19.0 (14.4-32) | 4.4 (2.6-5.6) | 290.0 (253.0-457.0) |
| [Niger](http://www.who.int/countries/ner/en/) | 24,206,636 | 15.2 | 63.6 | 0.92 (0.83-1.37) | 0.3 (0.0-17.0) | 0.0 (0.0-0.4) | 0.3 (0.0-14.4) | 0.1 (0.0-2.5) | 697.5 (624.0-700^b^) |
| [Nigeria](http://www.who.int/countries/nga/en/) | 206,139,587 | 18.1 | 55.8 | 1.03 (0.95-1.57) | 2.5 (0.0-28.6) | 0.1 (0.0-0.6) | 2.5 (0.0-24.4) | 0.5 (0.0-4.3) | 705.0 (459.0-700^b^) |
| [Rwanda](http://www.who.int/countries/rwa/en/) | 12,952,209 | 20.0 | 70.0 | 1.15 (1.03-1.69) | 25.0 (6.5-33.5) | 0.6 (0.2-0.8) | 19.0 (5.5-28.5) | 3.7 (1.0-5.0) | 405.0 (320.0-700^b^) |
| [Senegal](http://www.who.int/countries/sen/en/) | 16,743,930 | 18.5 | 68.9 | 1.05 (0.96-1.59) | 22.5 (0.0-29.0) | 0.6 (0.0-0.7) | 1.5 (0.0-24.7) | 3.3 (0.0-4.3) | 675.0 (382.0-700^b^) |
| [Sierra Leone](http://www.who.int/countries/sle/en/) | 7,976,985 | 19.4 | 55.9 | 1.05 (0.99-1.64) | 21.0 (0.4-31.1) | 0.5 (0.0-0.7) | 19.0 (0.4-26.5) | 3.2 (0.1-4.6) | 425.0 (338.0-700^b^) |
| [South Africa](http://www.who.int/countries/zaf/en/) | 59,308,690 | 27.6 | 64.9 | 1.45 (1.32-2.16) | 45.0 (33.7-52.0) | 1.2 (1.0-1.4) | 37.0 (28.4-44.0) | 7.1 (5.4-8.0) | 232.5 (217.0-268.0) |
| [South Sudan](http://www.who.int/countries/ssd/en/) | 11,193,729 | 19.0 | 58.7 | 1.05 (0.98-1.62) | 21.0 (0.2-30.3) | 0.6 (0.0-0.7) | 17.0 (0.1-25.8) | 3.2 (0.0-4.5) | 675.0 (353.0-700^b^) |
| [Togo](http://www.who.int/countries/tgo/en/) | 8,278,737 | 19.4 | 62.1 | 1.05 (1.00-1.65) | 23.0 (1.1-31.6) | 0.6 (0.0-0.7) | 19.0 (1.0-27.0) | 3.4 (0.2-4.7) | 375.0 (330.0-700^b^) |
| [Uganda](http://www.who.int/countries/uga/en/) | 45,741,000 | 16.7 | 64.4 | 0.92 (0.86-1.42) | 0.5 (0.0-22.0) | 0.0 (0.0-0.4) | 0.5 (0.0-18.8) | 0.1 (0.0-3.2) | 697.5 (575.0-700^b^) |
| [Tanzania](http://www.who.int/countries/tza/en/) | 59,734,213 | 18.0 | 66.4 | 0.98 (0.94-1.55) | 2.5 (0.0-27.7) | 0.1 (0.0-0.6) | 2.5 (0.0-23.6) | 0.5 (0.0-4.1) | 705.0 (443.0-700^b^) |
| [Zambia](http://www.who.int/countries/zmb/en/) | 18,383,956 | 17.6 | 64.7 | 1.03 (0.89-1.48) | 2.5 (0.0-24.9) | 0.1 (0.0-0.5) | 1.5 (0.0-21.3) | 0.3 (0.0-3.6) | 690.0 (472.0-700^b^) |
| [Zimbabwe](http://www.who.int/countries/zwe/en/) | 14,862,927 | 18.7 | 62.2 | 1.05 (0.97-1.60) | 22.5 (0.0-29.3) | 0.6 (0.0-0.7) | 16.5 (0.0-25.0) | 3.3 (0.0-4.3) | 675.0 (377.0-700^b^) |
| AFRO | 1,118,418,151 | 18.9 | 63.3^c^ | 1.05 (0.93-1.95)^c^ | 22.5 (0.25-63.5)^c^ | 0.5 (0.0-2.8)^c^ | 17.0 (0.25-52.5)^c^ | 3.3 (0.0-11.3)^c^ | 675 (116.0-700^b^)^c^ |

Cent Afr Rep: Central African Republic; D Rep Congo: [Democratic Republic of the Congo](http://www.who.int/countries/cod/en/); [Equ Guinea](http://www.who.int/countries/gnq/en/): [Equatorial Guinea](http://www.who.int/countries/gnq/en/); N: number; NA: not applicable; [Tanzania](http://www.who.int/countries/tza/en/): [United Republic of Tanzania](http://www.who.int/countries/tza/en/); UI: uncertainty interval.

^a^For each epidemiologic indicator, the table reports the most probable estimate and the 95% uncertainty interval. For a number of countries, *R*_0_ was close to 1 in some of the uncertainty runs leading to an estimated time for peak incidence beyond the simulation duration of two years (delayed epidemic peak). For these countries, the upper limit of the uncertainty interval was set at 700 days, that is the end day of the simulation. Uncertainty runs in which *R*_0_ was less than 1 led to no epidemic emergence, and thus were excluded from further analysis. Reported ranges for the epidemic indicators did not include these runs, except for the range of *R*_0_.

^b^The estimated time for peak incidence was beyond the simulation duration of two years. The upper limit of the uncertainty interval was set at 700 days, that is the end day of the simulation.

^c^Estimates are for the median (and range).

**S4 Table. Model estimates for key SARS-CoV-2 epidemiologic indicators for countries and territories with a population of at least one million [7] in the World Health Organization Region of the Americas (AMRO).**

| Region of the Americas | Total population [7] | Med-ian age [7] | Life expec-tancy [7] | *R*_0_^a^ | Infections per 100 persons^a^ | Deaths per 100 persons^a^ | Mild infections per 100 persons^a^ | Severe and/or critical disease cases per 100 persons^a^ | Day at peak incidence^a^ |
| --- | --- | --- | --- | --- | --- | --- | --- | --- | --- |
| Country | N | Years | Years | N (95% UI) | N (95% UI) | N (95% UI) | N (95% UI) | N (95% UI) | N (95% UI) |
| Argentina | 45,195,777 | 31.5 | 77.2 | 1.65 (1.51-2.47) | 53.0 (44.8-60.0) | 2.3 (2.0-2.5) | 45.5 (37.0-49.8) | 9.1 (7.8-10.2) | 180.5 (174.0-188.0) |
| Bolivia | 11,673,029 | 25.6 | 72.4 | 1.45 (1.28-2.10) | 41.0 (30.3-48.6) | 1.5 (1.2-1.6) | 33.0 (25.3-40.8) | 6.8 (5.0-7.8) | 227.5 (207.0-262.0) |
| [Brazil](http://www.who.int/countries/bra/en/) | 212,559,409 | 33.5 | 76.6 | 1.75 (1.57-2.57) | 57.0 (49.3-64.3) | 2.1 (2.0-2.4) | 49.5 (41.0-53.7) | 9.7 (8.4-10.6) | 183.5 (180.0-190.0) |
| [Canada](http://www.who.int/countries/can/en/) | 37,742,157 | 41.1 | 83.0 | 2.05 (1.86-3.02) | 69.5 (63.0-74.5) | 3.7 (3.6-4.1) | 56.5 (51.1-60.7) | 13.1 (11.9-13.8) | 131.3 (127.0-133.0) |
| [Chile](http://www.who.int/countries/chl/en/) | 19,116,209 | 35.3 | 80.7 | 1.85 (1.66-2.71) | 61.5 (54.0-67.7) | 2.6 (2.4-2.9) | 51.5 (44.5-56.1) | 10.7 (9.5-11.6) | 146.9 (145.0-148.0) |
| [Colombia](http://www.who.int/countries/col/en/) | 50,882,884 | 31.3 | 77.9 | 1.65 (1.50-2.46) | 55.0 (45.4-61.1) | 2.0 (1.8-2.2) | 45.5 (37.7-51.1) | 9.1 (7.7-10.0) | 182.5 (176.0-191.0) |
| [Costa Rica](http://www.who.int/countries/cri/en/) | 5,094,114 | 33.5 | 80.9 | 1.75 (1.58-2.59) | 59.0 (49.9-64.6) | 2.3 (2.1-2.5) | 48.5 (41.4-53.8) | 9.9 (8.6-10.8) | 144.3 (142.0-148.0) |
| [Cuba](http://www.who.int/countries/cub/en/) | 11,326,616 | 42.2 | 79.2 | 2.05 (1.84-2.99) | 68.5 (62.4-74.1) | 3.3 (3.2-3.7) | 56.5 (50.9-60.7) | 12.5 (11.5-13.4) | 122.3 (120.0-125.0) |
| [Dom. Rep.](http://www.who.int/countries/dom/en/) | 10,847,904 | 28.0 | 74.7 | 1.45 (1.37-2.24) | 47.0 (36.5-53.8) | 1.6 (1.4-1.8) | 39.0 (30.4-45.1) | 7.7 (6.1-8.7) | 196.5 (184.0-215.0) |
| [Ecuador](http://www.who.int/countries/ecu/en/) | 17,643,060 | 27.9 | 77.7 | 1.45 (1.36-2.24) | 47.0 (36.4-53.7) | 1.7 (1.4-1.8) | 39.0 (30.4-45.1) | 7.7 (6.0-8.6) | 202.5 (191.0-224.0) |
| [El Salvador](http://www.who.int/countries/slv/en/) | 6,486,201 | 27.6 | 74.1 | 1.45 (1.37-2.25) | 47.0 (36.8-54.0) | 1.7 (1.5-1.9) | 39.0 (30.6-45.2) | 7.7 (6.2-8.8) | 187.5 (178.0-207.0) |
| [Guatemala](http://www.who.int/countries/gtm/en/) | 17,915,567 | 22.9 | 75.1 | 1.25 (1.14-1.88) | 35.0 (21.3-41.3) | 1.0 (0.7-1.1) | 29.0 (18.0-35.1) | 5.0 (3.4-6.3) | 305.0 (262.0-410.0) |
| Haiti | 11,402,533 | 24.0 | 65.0 | 1.35 (1.19-1.96) | 35.0 (24.7-44.3) | 1.1 (0.8-1.2) | 31.0 (20.8-37.5) | 5.6 (3.9-6.8) | 265.0 (233.0-332.0) |
| [Honduras](http://www.who.int/countries/hnd/en/) | 9,904,608 | 24.3 | 75.9 | 1.35 (1.20-1.98) | 37.0 (25.8-45.6) | 1.0 (0.8-1.2) | 31.0 (21.7-38.7) | 5.9 (4.1-6.9) | 255.0 (227.0-318.0) |
| [Jamaica](http://www.who.int/countries/jam/en/) | 2,961,161 | 30.7 | 74.9 | 1.65 (1.48-2.43) | 53.0 (43.9-59.9) | 2.0 (1.8-2.2) | 45.5 (36.5-50.0) | 9.1 (7.5-9.9) | 155.5 (150.0-162.0) |
| [Mexico](http://www.who.int/countries/mex/en/) | 128,932,753 | 29.2 | 75.4 | 1.55 (1.41-2.31) | 49.0 (39.5-56.2) | 1.7 (1.5-1.9) | 40.5 (32.9-47.2) | 8.0 (6.5-9.1) | 214.5 (204.0-234.0) |
| [Nicaragua](http://www.who.int/countries/nic/en/) | 6,624,554 | 26.5 | 75.2 | 1.45 (1.28-2.11) | 41.0 (31.1-49.8) | 1.2 (1.0-1.4) | 35.0 (26.1-42.1) | 6.5 (5.0-7.7) | 212.5 (198.0-250.0) |
| [Panama](http://www.who.int/countries/pan/en/) | 4,314,768 | 29.7 | 79.1 | 1.55 (1.42-2.34) | 49.0 (39.9-56.4) | 1.8 (1.6-2.0) | 40.5 (33.2-47.2) | 8.3 (6.7-9.2) | 171.0 (163.0-182.0) |
| [Paraguay](http://www.who.int/countries/pry/en/) | 7,132,530 | 26.3 | 74.6 | 1.45 (1.29-2.13) | 43.0 (31.8-50.3) | 1.4 (1.1-1.6) | 35.0 (26.6-42.3) | 7.1 (5.2-8) | 212.5 (196.0-244.0) |
| [Peru](http://www.who.int/countries/per/en/) | 32,971,846 | 31.0 | 77.4 | 1.65 (1.47-2.41) | 53.0 (43.3-59.4) | 2.0 (1.7-2.1) | 43.5 (36.1-49.7) | 8.7 (7.3-9.7) | 185.0 (177.0-195.0) |
| Puerto Rico | 2,860,840 | 44.5 | 80.7 | 2.28 (1.99-2.61) | 70.5 (64.0-74.7) | 4.2 (4.0-4.6) | 56.5 (51.5-60.4) | 13.5 (12.5-14.3) | 106.3 (103.0-110.0) |
| [Trin. and Tob.](http://www.who.int/countries/tto/en/) | 1,399,491 | 36.2 | 73.9 | 1.85 (1.66-2.72) | 61.5 (54.0-67.6) | 2.5 (2.3-2.8) | 51.5 (44.5-56.1) | 10.7 (9.4-11.6) | 121.9 (121.0-123.0) |
| [USA](http://www.who.int/countries/usa/en/) | 331,002,647 | 38.3 | 79.1 | 1.95 (1.76-2.86) | 64.5 (57.9-70.2) | 3.4 (3.2-3.8) | 52.5 (47.1-57.4) | 12.1 (10.9-12.9) | 158.9 (156.0-160.0) |
| [Uruguay](http://www.who.int/countries/ury/en/) | 3,473,727 | 35.8 | 78.4 | 1.85 (1.66-2.71) | 60.5 (53.3-66.6) | 3.2 (2.8-3.3) | 49.5 (43.5-54.8) | 10.9 (9.7-11.9) | 130.9 (129.0-132.0) |
| Venezuela | 28,435,943 | 29.6 | 72.3 | 1.55 (1.41-2.32) | 49.0 (39.2-55.5) | 1.8 (1.5-2.0) | 40.5 (32.6-46.4) | 8.0 (6.6-9.1) | 195.0 (185.0-210.0) |
| AMRO | 1,017,900,328 | 30.7 | 76.6^c^ | 1.65 (1.25-2.28)^c^ | 53.0 (35.0-70.5)^c^ | 2.0 (1.0-4.2)^c^ | 43.5 (29.0-56.5)^c^ | 8.7 (5.0-13.5)^c^ | 183.5 (106.3-305.0)^c^ |

Dom Rep: Dominican Republic; N: number; [Trin. and Tob.:](http://www.who.int/countries/tto/en/) [Trinidad and Tobago](http://www.who.int/countries/tto/en/); UI: uncertainty interval; USA: United States of America.

^a^For each epidemiologic indicator, the table reports the most probable estimate and the 95% uncertainty interval. For a number of countries, *R*_0_ was close to 1 in some of the uncertainty runs leading to an estimated time for peak incidence beyond the simulation duration of two years (delayed epidemic peak). For these countries, the upper limit of the uncertainty interval was set at 700 days, that is the end day of the simulation. Uncertainty runs in which *R*_0_ was less than 1 led to no epidemic emergence, and thus were excluded from further analysis. Reported ranges for the epidemic indicators did not include these runs, except for the range of *R*_0_.

^c^Estimates are for the median (and range).

**S5 Table. Model estimates for key SARS-CoV-2 epidemiologic indicators for countries and territories with a population of at least one million [7] in the World Health Organization Eastern Mediterranean Region (EMRO).**

| Eastern Mediterranean Region | Total population [7] | Med-ian age [7] | Life expec-tancy [7] | *R*_0_^a^ | Infections per 100 persons^a^ | Deaths per 100 persons^a^ | Mild infections per 100 persons^a^ | Severe and/or critical disease cases per 100 persons^a^ | Day at peak incidence^a^ |
| --- | --- | --- | --- | --- | --- | --- | --- | --- | --- |
| Country | N | Years | Years | N (95% UI) | N (95% UI) | N (95% UI) | N (95% UI) | N (95% UI) | N (95% UI) |
| [Afghanistan](http://www.who.int/countries/afg/en/) | 38,928,341 | 18.4 | 66.0 | 1.03 (0.94-1.55) | 2.5 (0.0-27.8) | 0.1 (0.0-0.6) | 2.5 (0.0-23.7) | 0.5 (0.0-4.1) | 700^b^ (436.0-700^b^) |
| [Bahrain](http://www.who.int/countries/bhr/en/) | 1,701,583 | 32.5 | 77.7 | 1.65 (1.50-2.48) | 61.0 (49.0-66.7) | 0.9 (0.8-1.1) | 51.0 (42.1-57.4) | 8.7 (7.0-9.4) | 146.5 (141.0-154.0) |
| Egypt | 102,334,403 | 24.6 | 72.5 | 1.35 (1.22-2.01) | 37.0 (26.7-45.5) | 1.2 (0.9-1.3) | 31.0 (22.4-38.4) | 5.9 (4.3-7.1) | 285.0 (255.0-350.0) |
| Iran | 83,992,953 | 32.0 | 77.3 | 1.55 (1.47-2.41) | 53.0 (43.9-60.2) | 1.7 (1.4-1.8) | 44.5 (36.8-50.8) | 8.7 (7.1-9.4) | 195.0 (187.0-207.0) |
| Iraq | 40,222,503 | 21.0 | 71.1 | 1.15 (1.06-1.75) | 27.0 (14.3-36.2) | 0.7 (0.4-0.8) | 23.0 (12.1-30.8) | 4.1 (2.2-5.4) | 375.0 (320.0-628.0) |
| Jordan | 10,203,140 | 23.8 | 75.0 | 1.25 (1.17-1.93) | 37.0 (23.5-43.5) | 0.9 (0.6-1.0) | 29.0 (19.9-37.0) | 5.3 (3.6-6.5) | 265.0 (238.0-353.0) |
| Kuwait | 4,270,563 | 36.8 | 75.9 | 1.75 (1.59-2.61) | 61.0 (51.6-67.0) | 1.2 (1.1-1.4) | 51.5 (43.9-57.1) | 9.1 (7.8-9.9) | 143.5 (139.0-147.0) |
| Lebanon | 6,825,442 | 29.6 | 79.3 | 1.55 (1.43-2.34) | 51.0 (40.5-57.2) | 1.8 (1.5-1.9) | 41.5 (33.8-48.0) | 8.3 (6.7-9.2) | 175.0 (168.0-188.0) |
| [Libya](http://www.who.int/countries/lby/en/) | 6,871,287 | 28.8 | 73.4 | 1.45 (1.34-2.20) | 45.0 (35.4-53.7) | 1.1 (0.9-1.2) | 39.0 (29.9-45.7) | 7.1 (5.4-8.0) | 196.5 (184.0-222.0) |
| [Morocco](http://www.who.int/countries/mar/en/) | 36,910,558 | 29.5 | 77.4 | 1.55 (1.42-2.33) | 49.0 (39.7-56.2) | 1.7 (1.5-1.9) | 40.5 (33.1-47.1) | 8.3 (6.7-9.2) | 197.0 (188.0-213.0) |
| [Oman](http://www.who.int/countries/omn/en/) | 5,106,622 | 30.6 | 78.6 | 1.55 (1.39-2.30) | 53.0 (41.9-61.4) | 0.7 (0.7-0.9) | 47.0 (36.1-53.0) | 7.4 (5.9-8.4) | 178.5 (170.0-199.0) |
| [Pakistan](http://www.who.int/countries/pak/en/) | 220,892,331 | 22.8 | 67.8 | 1.25 (1.14-1.88) | 33.0 (21.8-41.3) | 0.9 (0.7-1.1) | 27.0 (18.3-35.0) | 5.0 (3.4-6.3) | 350.0 (303.0-479.0) |
| Palestine | 5,101,416 | 20.8 | 74.6 | 1.15 (1.04-1.71) | 25.0 (12.3-34.5) | 0.6 (0.3-0.8) | 19.0 (10.4-29.4) | 3.8 (1.9-5.1) | 345.0 (293.0-627.0) |
| [Qatar](http://www.who.int/countries/qat/en/) | 2,881,060 | 32.3 | 80.7 | 1.65 (1.54-2.55) | 65.0 (53.6-71.5) | 0.8 (0.7-0.9) | 55.0 (46.3-61.8) | 8.9 (7.4-9.7) | 147.5 (141.0-153.0) |
| [Saudi Arabia](http://www.who.int/countries/sau/en/) | 34,813,867 | 31.8 | 75.7 | 1.55 (1.42-2.35) | 53.0 (42.1-59.7) | 1.0 (0.9-1.1) | 45.0 (35.9-51.1) | 7.9 (6.2-8.7) | 193.5 (185.0-212.0) |
| [Somalia](http://www.who.int/countries/som/en/) | 15,893,219 | 16.7 | 58.3 | 0.98 (0.88-1.45) | 1.5 (0.0-22.8) | 0.0 (0.0-0.5) | 1.5 (0.0-19.4) | 0.3 (0.0-3.4) | 697.5 (501.0-700^b^) |
| Sudan | 43,849,269 | 19.7 | 66.1 | 1.15 (1.02-1.68) | 23.0 (1.7-32.9) | 0.7 (0.0-0.8) | 21.0 (1.5-27.9) | 3.7 (0.3-5.0) | 405.0 (352.0-700^b^) |
| [Syria](http://www.who.int/countries/syr/en/) | 17,500,657 | 25.6 | 76.1 | 1.35 (1.24-2.05) | 39.0 (28.6-48.0) | 1.1 (0.8-1.2) | 33.0 (24.2-40.7) | 6.2 (4.5-7.3) | 242.5 (222.0-295.0) |
| [Tunisia](http://www.who.int/countries/tun/en/) | 11,818,618 | 32.8 | 77.4 | 1.65 (1.53-2.50) | 55.0 (46.3-61.5) | 2.0 (1.8-2.2) | 45.5 (38.4-51.3) | 9.5 (7.8-10.2) | 162.5 (158.0-168.0) |
| [UAE](http://www.who.int/countries/are/en/) | 9,890,400 | 32.6 | 78.5 | 1.65 (1.53-2.54) | 63.0 (52.8-70.6) | 0.7 (0.6-0.8) | 55.0 (45.6-61.2) | 8.7 (7.2-9.5) | 161.0 (154.0-169.0) |
| [Yemen](http://www.who.int/countries/yem/en/) | 29,825,968 | 20.2 | 66.4 | 1.05 (1.00-1.66) | 23.0 (0.4-32.8) | 0.6 (0.0-0.7) | 19.0 (0.4-28.0) | 3.4 (0.1-4.8) | 425.0 (357.0-700^b^) |
| EMRO | 729,834,200 | 28.8 | 75.7^c^ | 1.45 (0.98-1.75)^c^ | 45.0 (1.5-65.0)^c^ | 0.9 (0.0-2.0)^c^ | 39.0 (1.5-55.0)^c^ | 7.1 (0.3-9.5)^c^ | 197.0 (143.5-700^b^)^c^ |

N: number; Syria: [Syrian Arab Republic](http://www.who.int/countries/syr/en/); UAE: [United Arab Emirates](http://www.who.int/countries/are/en/); UI: uncertainty interval.

^a^For each epidemiologic indicator, the table reports the most probable estimate and the 95% uncertainty interval. For a number of countries, *R*_0_ was close to 1 in some of the uncertainty runs leading to an estimated time for peak incidence beyond the simulation duration of two years (delayed epidemic peak). For these countries, the upper limit of the uncertainty interval was set at 700 days, that is the end day of the simulation. Uncertainty runs in which *R*_0_ was less than 1 led to no epidemic emergence, and thus were excluded from further analysis. Reported ranges for the epidemic indicators did not include these runs, except for the range of *R*_0_.

^b^The estimated time for peak incidence was beyond the simulation duration of two years. The upper limit of the uncertainty interval was set at 700 days, that is the end day of the simulation.

^c^Estimates are for the median (and range).

**S6 Table. Model estimates for key SARS-CoV-2 epidemiologic indicators for countries and territories with a population of at least one million [7] in the World Health Organization European Region (EURO).**

| European Region | Total population [7] | Med-ian age [7] | Life expec-tancy [7] | *R*_0_^a^ | Infections per 100 persons^a^ | Deaths per 100 persons^a^ | Mild infections per 100 persons^a^ | Severe and/or critical disease cases per 100 persons^a^ | Day at peak incidence^a^ |
| --- | --- | --- | --- | --- | --- | --- | --- | --- | --- |
| Country | N | Years | Years | N (95% UI) | N (95% UI) | N (95% UI) | N (95% UI) | N (95% UI) | N (95% UI) |
| Albania | 2,877,800 | 36.4 | 79.0 | 1.85 (1.73-2.82) | 63.5 (57.1-69.9) | 3.1 (2.9-3.5) | 52.5 (46.6-57.3) | 11.7 (10.5-12.6) | 122.0 (120.0-123.0) |
| Armenia | 2,963,234 | 35.4 | 75.6 | 1.85 (1.67-2.72) | 61.5 (53.8-67.4) | 2.7 (2.5-3.0) | 50.5 (44.2-55.7) | 10.9 (9.6-11.8) | 128.9 (127.0-130.0) |
| Austria | 9,006,400 | 43.5 | 82.1 | 2.05 (1.91-3.11) | 71.5 (65.7-76.6) | 4.0 (3.8-4.4) | 58.5 (53.2-62.3) | 13.5 (12.5-14.3) | 114.3 (111.0-118.0) |
| [Azerbaijan](http://www.who.int/countries/aze/en/) | 10,139,175 | 32.3 | 73.3 | 1.65 (1.52-2.49) | 55.0 (46.5-62.1) | 1.8 (1.6-2.0) | 46.5 (38.8-52.1) | 9.3 (7.7-10.0) | 161.5 (157.0-168.0) |
| [Belarus](http://www.who.int/countries/blr/en/) | 9,449,321 | 40.3 | 75.2 | 2.05 (1.84-2.99) | 68.5 (62.2-73.8) | 3.4 (3.2-3.7) | 55.5 (50.7-60.4) | 12.7 (11.5-13.4) | 121.3 (119.0-124.0) |
| [Belgium](http://www.who.int/countries/bel/en/) | 11,589,616 | 41.9 | 82.2 | 2.05 (1.85-3.01) | 68.5 (62.1-73.5) | 4.1 (3.7-4.3) | 55.5 (50.2-59.7) | 13.1 (11.9-13.8) | 121.3 (119.0-124.0) |
| [Bosnia & Herz.](http://www.who.int/countries/bih/en/) | 3,280,815 | 43.1 | 77.9 | 2.05 (1.91-3.11) | 71.5 (65.5-76.5) | 3.8 (3.6-4.2) | 58.5 (53.1-62.3) | 13.5 (12.4-14.2) | 108.3 (104.0-110.0) |
| [Bulgaria](http://www.who.int/countries/bgr/en/) | 6,948,445 | 44.6 | 75.5 | 2.15 (1.96-3.19) | 71.5 (67.3-77.7) | 4.3 (4.1-4.6) | 58.5 (54.3-63.0) | 14.1 (13.0-14.8) | 110.5 (105.0-113.0) |
| [Croatia](http://www.who.int/countries/hrv/en/) | 4,105,268 | 44.3 | 79.0 | 2.15 (1.95-3.16) | 72.5 (66.6-77.2) | 4.3 (4.1-4.7) | 58.5 (53.7-62.4) | 14.1 (13.0-14.7) | 106.5 (103.0-110.0) |
| [Cyprus](http://www.who.int/countries/cyp/en/) | 1,207,361 | 37.3 | 81.5 | 1.85 (1.74-2.84) | 65.5 (58.2-71.2) | 3.0 (2.8-3.3) | 55.5 (47.8-58.8) | 11.7 (10.4-12.5) | 113 (112.0-115.0) |
| [Czech Republic](http://www.who.int/countries/cze/en/) | 10,708,982 | 43.2 | 79.9 | 2.05 (1.92-3.11) | 71.5 (65.4-76.3) | 3.9 (3.7-4.3) | 57.5 (53.0-62.1) | 13.3 (12.4-14.2) | 115.3 (112.0-119.0) |
| [Denmark](http://www.who.int/countries/dnk/en/) | 5,792,203 | 42.3 | 81.4 | 2.05 (1.85-3.01) | 68.5 (62.3-73.6) | 4.0 (3.8-4.3) | 55.5 (50.3-59.7) | 13.1 (12.0-13.9) | 117.3 (113.0-119.0) |
| [Estonia](http://www.who.int/countries/est/en/) | 1,326,539 | 42.4 | 79.2 | 2.05 (1.89-3.07) | 71.5 (64.1-75.1) | 4.0 (3.8-4.4) | 57.5 (51.8-61.0) | 13.3 (12.3-14.1) | 103.3 (99.0-105.0) |
| [Finland](http://www.who.int/countries/fin/en/) | 5,540,718 | 43.1 | 82.5 | 2.05 (1.91-3.09) | 71.5 (64.3-75.1) | 4.3 (4.1-4.8) | 56.5 (51.6-60.6) | 13.7 (12.7-14.5) | 111.3 (108.0-115.0) |
| [France](http://www.who.int/countries/fra/en/) | 65,273,512 | 42.3 | 83.1 | 2.05 (1.85-3.00) | 67.5 (61.5-72.7) | 4.0 (3.8-4.4) | 55.5 (49.6-58.9) | 12.9 (12.0-13.9) | 136.3 (132.0-138.0) |
| [Georgia](http://www.who.int/countries/geo/en/) | 3,989,175 | 38.3 | 74.2 | 1.85 (1.74-2.83) | 63.5 (57.0-69.5) | 3.2 (3.0-3.5) | 52.5 (46.4-56.9) | 11.7 (10.5-12.6) | 122.9 (122.0-125.0) |
| [Germany](http://www.who.int/countries/deu/en/) | 83,783,945 | 45.7 | 81.9 | 2.15 (1.97-3.19) | 73.5 (67.5-77.8) | 4.5 (4.3-4.9) | 59.5 (54.3-62.9) | 14.1 (13.2-15.0) | 127.5 (122.0-130.0) |
| [Greece](http://www.who.int/countries/grc/en/) | 10,423,056 | 45.6 | 82.8 | 2.15 (1.98-3.21) | 73.5 (68.1-78.3) | 4.5 (4.3-4.9) | 59.5 (54.8-63.3) | 14.1 (13.2-15.0) | 112.5 (107.0-115.0) |
| [Hungary](http://www.who.int/countries/hun/en/) | 9,660,350 | 43.3 | 77.3 | 2.05 (1.92-3.13) | 71.5 (66.0-76.8) | 4.0 (3.8-4.4) | 58.5 (53.4-62.5) | 13.7 (12.6-14.3) | 115.3 (111.0-118.0) |
| [Ireland](http://www.who.int/countries/irl/en/) | 4,937,796 | 38.2 | 82.8 | 1.85 (1.70-2.77) | 60.5 (55.1-68.0) | 2.9 (2.7-3.2) | 49.5 (45.1-56.0) | 11.1 (10.0-12.0) | 128.8 (128.0-130.0) |
| [Israel](http://www.who.int/countries/isr/en/) | 8,655,541 | 30.5 | 83.5 | 1.65 (1.47-2.40) | 49.0 (41.6-56.9) | 2.4 (2.1-2.6) | 41.5 (34.2-47.1) | 8.9 (7.5-9.9) | 169.5 (163.0-178.0) |
| [Italy](http://www.who.int/countries/ita/en/) | 60,461,828 | 47.3 | 84.0 | 2.30 (2.02-3.27) | 75.5 (69.6-79.5) | 4.7 (4.5-5.1) | 60.5 (55.9-64.1) | 14.7 (13.7-15.4) | 121.5 (116.0-124.0) |
| [Kazakhstan](http://www.who.int/countries/kaz/en/) | 18,776,707 | 30.7 | 73.9 | 1.55 (1.44-2.36) | 49.0 (40.6-56.6) | 1.9 (1.6-2.0) | 41.5 (33.8-47.3) | 8.3 (6.9-9.3) | 185.0 (176.0-197.0) |
| [Kyrgyzstan](http://www.who.int/countries/kgz/en/) | 6,524,191 | 26.0 | 72.0 | 1.35 (1.26-2.07) | 39.0 (29.1-47.9) | 1.2 (0.9-1.3) | 33.0 (24.5-40.5) | 6.2 (4.7-7.4) | 222.5 (203.0-263.0) |
| [Latvia](http://www.who.int/countries/lva/en/) | 1,886,202 | 43.9 | 75.7 | 2.05 (1.92-3.11) | 69.5 (65.1-75.8) | 4.4 (4.0-4.6) | 57.5 (52.5-61.4) | 13.7 (12.6-14.4) | 103.3 (99.0-106.0) |
| [Lithuania](http://www.who.int/countries/ltu/en/) | 2,722,291 | 45.1 | 76.4 | 2.05 (1.93-3.13) | 71.5 (65.9-76.5) | 4.2 (4.0-4.6) | 57.5 (53.1-61.9) | 13.9 (12.8-14.6) | 104.3 (101.0-108.0) |
| [Netherlands](http://www.who.int/countries/nld/en/) | 17,134,873 | 43.3 | 82.8 | 2.05 (1.88-3.06) | 69.5 (63.5-74.5) | 4.0 (3.8-4.4) | 56.5 (51.3-60.4) | 13.5 (12.3-14.1) | 121.3 (118.0-125.0) |
| [Norway](http://www.who.int/countries/nor/en/) | 5,421,242 | 39.8 | 82.9 | 1.95 (1.80-2.92) | 66.5 (60.1-72.1) | 3.7 (3.3-3.9) | 54.5 (48.9-58.9) | 12.3 (11.2-13.2) | 120.3 (118.0-123.0) |
| [Poland](http://www.who.int/countries/pol/en/) | 37,846,605 | 41.7 | 79.3 | 2.05 (1.90-3.09) | 71.5 (65.1-76.2) | 3.9 (3.7-4.2) | 58.5 (52.8-62.1) | 13.3 (12.3-14.1) | 127.3 (123.0-129.0) |
| [Portugal](http://www.who.int/countries/prt/en/) | 10,196,707 | 46.2 | 82.7 | 2.30 (1.99-3.24) | 74.5 (68.7-78.8) | 4.5 (4.3-4.9) | 60.5 (55.3-63.6) | 14.5 (13.4-15.1) | 109.5 (106.0-113.0) |
| [Rep. Moldova](http://www.who.int/countries/mda/en/) | 4,033,963 | 37.6 | 72.3 | 1.95 (1.78-2.91) | 67.5 (60.8-73.4) | 2.8 (2.6-3.1) | 56.5 (50.1-60.8) | 12.1 (10.7-12.6) | 119.3 (118.0-122.0) |
| [Romania](http://www.who.int/countries/rou/en/) | 19,237,682 | 43.2 | 76.5 | 2.05 (1.90-3.09) | 71.5 (64.9-75.8) | 4.0 (3.7-4.3) | 57.5 (52.5-61.7) | 13.5 (12.3-14.1) | 120.3 (117.0-124.0) |
| [Russia](http://www.who.int/countries/rus/en/) | 145,934,460 | 39.6 | 73.0 | 1.95 (1.81-2.95) | 67.5 (60.8-72.6) | 3.3 (3.2-3.7) | 54.5 (49.6-59.5) | 12.3 (11.2-13.1) | 145.3 (143.0-147.0) |
| Serbia | 8,737,370 | 41.6 | 76.5 | 2.05 (1.87-3.03) | 69.5 (63.2-74.6) | 3.8 (3.6-4.1) | 56.5 (51.3-60.7) | 13.1 (12.0-13.8) | 119.3 (115.0-121.0) |
| [Slovakia](http://www.who.int/countries/svk/en/) | 5,459,643 | 41.2 | 78.0 | 2.05 (1.87-3.04) | 71.5 (63.9-75.4) | 3.5 (3.3-3.8) | 57.5 (52.2-61.8) | 12.9 (11.8-13.6) | 114.3 (112.0-117.0) |
| [Slovenia](http://www.who.int/countries/svn/en/) | 2,078,932 | 44.5 | 81.8 | 2.15 (1.96-3.17) | 72.5 (67.0-77.3) | 4.2 (4.0-4.6) | 59.5 (54.0-62.7) | 13.9 (12.9-14.7) | 102.3 (97.0-104.0) |
| [Spain](http://www.who.int/countries/esp/en/) | 46,754,783 | 44.9 | 84.0 | 2.15 (1.95-3.17) | 71.5 (67.2-77.7) | 4.1 (3.9-4.5) | 59.5 (54.4-63.2) | 13.9 (12.8-14.5) | 123.5 (119.0-127.0) |
| [Sweden](http://www.who.int/countries/swe/en/) | 10,099,270 | 41.1 | 83.3 | 1.95 (1.83-2.98) | 67.5 (61.3-72.8) | 4.0 (3.7-4.3) | 55.5 (49.5-59.1) | 12.9 (11.8-13.7) | 122.3 (119.0-125.0) |
| [Switzerland](http://www.who.int/countries/che/en/) | 8,654,618 | 43.1 | 84.2 | 2.05 (1.9-3.09) | 71.5 (65.2-76.1) | 3.9 (3.7-4.3) | 58.5 (52.8-62.0) | 13.5 (12.4-14.2) | 116.3 (112.0-118.0) |
| [Tajikistan](http://www.who.int/countries/tjk/en/) | 9,537,642 | 22.4 | 71.8 | 1.25 (1.12-1.84) | 31.0 (19.4-39.5) | 0.8 (0.5-0.9) | 25.0 (16.4-33.6) | 4.7 (3.0-5.9) | 310 (260.0-431.0) |
| [Macedonia](http://www.who.int/countries/mkd/en/) | 2,083,380 | 39.1 | 76.3 | 1.95 (1.79-2.92) | 67.5 (60.5-72.8) | 3.1 (2.9-3.4) | 55.5 (49.6-59.9) | 12.1 (10.9-12.9) | 113.3 (111.0-115.0) |
| [Turkey](http://www.who.int/countries/tur/en/) | 84,339,067 | 31.5 | 78.5 | 1.65 (1.49-2.44) | 55.0 (44.3-60.0) | 2.0 (1.7-2.2) | 44.5 (36.8-50.1) | 9.1 (7.5-9.9) | 189 (183.0-200.0) |
| [Turkmenistan](http://www.who.int/countries/tkm/en/) | 6,031,187 | 26.9 | 68.6 | 1.45 (1.29-2.12) | 41.0 (31.2-49.8) | 1.2 (0.9-1.3) | 37.0 (26.2-42.1) | 6.5 (5.0-7.7) | 212.5 (195.0-245.0) |
| [Ukraine](http://www.who.int/countries/ukr/en/) | 43,733,759 | 41.2 | 72.5 | 2.05 (1.87-3.05) | 70.5 (64.1-75.5) | 3.6 (3.4-3.9) | 57.5 (52.2-61.8) | 12.9 (11.9-13.7) | 129.3 (127.0-132.0) |
| [United Kingdom](http://www.who.int/countries/gbr/en/) | 67,886,004 | 40.5 | 81.8 | 2.05 (1.82-2.95) | 65.5 (60.8-72.5) | 3.8 (3.5-4.1) | 54.5 (49.3-59.1) | 12.5 (11.5-13.4) | 138.3 (136.0-141.0) |
| [Uzbekistan](http://www.who.int/countries/uzb/en/) | 33,469,199 | 27.8 | 72.0 | 1.45 (1.33-2.18) | 45.0 (34.4-52.6) | 1.2 (1.0-1.4) | 37.0 (28.9-44.6) | 7.1 (5.5-8.1) | 222.5 (207.0-253.0) |
| EURO | 930,700,857 | 41.7 | 79.0^c^ | 2.05 (1.25-2.30)^c^ | 69.0 (31.0-75.5)^c^ | 3.9 (0.8-4.7)^c^ | 56.5 (25.0-65.0)^c^ | 13.0 (4.7-14.7)^c^ | 121.3 (102.3-310.0)^c^ |

[Bosnia & Herz:](http://www.who.int/countries/bih/en/) [Bosnia and Herzegovina](http://www.who.int/countries/bih/en/); Macedonia: [Republic of North Macedonia](http://www.who.int/countries/mkd/en/); N: number; [Rep Moldova](http://www.who.int/countries/mda/en/): [Republic of Moldova](http://www.who.int/countries/mda/en/); Russia: [Russian Federation](http://www.who.int/countries/rus/en/); UI: uncertainty interval.

^a^For each epidemiologic indicator, the table reports the most probable estimate and the 95% uncertainty interval. For a number of countries, *R*_0_ was close to 1 in some of the uncertainty runs leading to an estimated time for peak incidence beyond the simulation duration of two years (delayed epidemic peak). For these countries, the upper limit of the uncertainty interval was set at 700 days, that is the end day of the simulation. Uncertainty runs in which *R*_0_ was less than 1 led to no epidemic emergence, and thus were excluded from further analysis. Reported ranges for the epidemic indicators did not include these runs, except for the range of *R*_0_.

^c^Estimates are for the median (and range).

**S7 Table. Model estimates for key SARS-CoV-2 epidemiologic indicators for countries and territories with a population of at least one million [7] in the World Health Organization South-East Asia Region (SEARO).**

| South-East Asia Region | Total population [7] | Med-ian age [7] | Life expec-tancy [7] | *R*_0_^a^ | Infections per 100 persons^a^ | Deaths per 100 persons^a^ | Mild infections per 100 persons^a^ | Severe and/or critical disease cases per 100 persons^a^ | Day at peak incidence^a^ |
| --- | --- | --- | --- | --- | --- | --- | --- | --- | --- |
| Country | N | Years | Years | N (95% UI) | N (95% UI) | N (95% UI) | N (95% UI) | N (95% UI) | N (95% UI) |
| [Bangladesh](http://www.who.int/countries/bgd/en/) | 164,689,383 | 27.6 | 73.6 | 1.45 (1.32-2.18) | 45.0 (34.6-53.0) | 1.2 (1.0-1.4) | 39.0 (29.2-44.9) | 7.1 (5.5-8.1) | 242.5 (228.0-281.0) |
| [DPR. Korea](http://www.who.int/countries/prk/en/) | 25,778,815 | 35.3 | 72.9 | 1.85 (1.63-2.66) | 61.5 (52.2-66.3) | 2.3 (2.1-2.6) | 49.5 (43.2-55.1) | 10.3 (9-11.2) | 154.3 (151.0-156.0) |
| [India](http://www.who.int/countries/ind/en/) | 1,380,004,385 | 28.4 | 70.4 | 1.45 (1.37-2.25) | 47.0 (37.7-55.0) | 1.6 (1.3-1.7) | 39.0 (31.6-46.3) | 7.7 (6.2-8.7) | 252.5 (240.0-283.0) |
| [Indonesia](http://www.who.int/countries/idn/en/) | 273,523,621 | 29.7 | 72.3 | 1.55 (1.41-2.32) | 49.0 (39.9-56.6) | 1.5 (1.3-1.7) | 41.5 (33.4-47.6) | 8.0 (6.5-9.0) | 223.5 (211.0-242.0) |
| [Myanmar](http://www.who.int/countries/mmr/en/) | 54,409,794 | 29.0 | 67.8 | 1.55 (1.39-2.29) | 49.0 (38.7-55.8) | 1.6 (1.3-1.7) | 41.0 (32.4-47.0) | 7.7 (6.3-8.8) | 208.5 (197.0-227.0) |
| [Nepal](http://www.who.int/countries/npl/en/) | 29,136,808 | 24.6 | 71.7 | 1.35 (1.24-2.04) | 39.0 (28.7-47.7) | 1.2 (1.0-1.4) | 33.0 (24.0-40.2) | 6.2 (4.6-7.5) | 252.5 (229.0-304.0) |
| [Sri Lanka](http://www.who.int/countries/lka/en/) | 21,413,250 | 34.0 | 77.6 | 1.75 (1.57-2.56) | 55.5 (47.8-62.2) | 2.4 (2.2-2.7) | 42.5 (39.3-51.4) | 9.7 (8.5-10.7) | 161.3 (158.0-165.0) |
| [Thailand](http://www.who.int/countries/tha/en/) | 69,799,978 | 40.1 | 77.7 | 1.95 (1.78-2.90) | 66.5 (59.7-72.2) | 2.9 (2.8-3.3) | 54.5 (49.0-59.5) | 11.9 (10.7-12.7) | 143.3 (141.0-145.0) |
| [Timor-Leste](http://www.who.int/countries/tls/en/) | 1,318,442 | 20.8 | 70.2 | 1.15 (1.06-1.75) | 25.0 (15.3-35.3) | 0.8 (0.5-1.0) | 23.0 (12.9-29.9) | 4.1 (2.5-5.4) | 290.0 (251.0-476.0) |
| SEARO | 2,020,074,476 | 29.0 | 72.3^c^ | 1.55 (1.15-1.95)^c^ | 49.0 (25.0-66.5)^c^ | 1.6 (0.8-2.9)^c^ | 41.0 (23.0-54.5)^c^ | 7.7 (4.1-11.9)^c^ | 223.5 (143.3-290.0)^c^ |

[DPR Korea](http://www.who.int/countries/prk/en/): [Democratic People's Republic of Korea](http://www.who.int/countries/prk/en/); N: number; UI: uncertainty interval.

^a^For each epidemiologic indicator, the table reports the most probable estimate and the 95% uncertainty interval. For a number of countries, *R*_0_ was close to 1 in some of the uncertainty runs leading to an estimated time for peak incidence beyond the simulation duration of two years (delayed epidemic peak). For these countries, the upper limit of the uncertainty interval was set at 700 days, that is the end day of the simulation. Uncertainty runs in which *R*_0_ was less than 1 led to no epidemic emergence, and thus were excluded from further analysis. Reported ranges for the epidemic indicators did not include these runs, except for the range of *R*_0_.

^c^Estimates are for the median (and range).

**S8 Table. Model estimates for key SARS-CoV-2 epidemiologic indicators for countries and territories with a population of at least one million [7] in the World Health Organization Western Pacific Region (WPRO).**

| Western Pacific Region | Total population [7] | Med-ian age [7] | Life expec-tancy [7] | *R*_0_^a^ | Infections per 100 persons^a^ | Deaths per 100 persons^a^ | Mild infections per 100 persons^a^ | Severe and/or critical disease cases per 100 persons^a^ | Day at peak incidence^a^ |
| --- | --- | --- | --- | --- | --- | --- | --- | --- | --- |
| Country | N | Years | Years | N (95% UI) | N (95% UI) | N (95% UI) | N (95% UI) | N (95% UI) | N (95% UI) |
| [Australia](http://www.who.int/countries/aus/en/) | 25,499,881 | 37.9 | 83.9 | 1.85 (1.74-2.83) | 63.5 (57.1-69.7) | 3.3 (3.1-3.6) | 52.5 (46.6-57.1) | 11.7 (10.6-12.6) | 139.9 (138.0-141.0) |
| [Cambodia](http://www.who.int/countries/khm/en/) | 16,718,971 | 25.6 | 70.5 | 1.35 (1.24-2.04) | 39.0 (28.2-47.6) | 1.1 (0.8-1.2) | 33.0 (23.8-40.3) | 6.2 (4.5-7.3) | 247.5 (223.0-299.0) |
| [China](http://www.who.int/countries/chn/en/) | 1,439,323,774 | 38.4 | 77.5 | 1.95 (1.75-2.86) | 65.5 (58.7-71.4) | 2.7 (2.5-3.0) | 54.5 (48.4-59.1) | 11.5 (10.3-12.3) | 170.9 (169.0-172.0) |
| Hong Kong | 7,496,988 | 44.8 | 85.3 | 2.30 (1.99-3.24) | 75.5 (69.7-80.1) | 4.1 (3.8-4.4) | 61.5 (56.5-65.2) | 14.1 (13.1-14.8) | 106.5 (104.0-111.0) |
| [Japan](http://www.who.int/countries/jpn/en/) | 126,476,458 | 48.4 | 85.0 | 2.30 (2.07-3.35) | 76.5 (71.2-80.6) | 5.3 (5.1-5.8) | 61.5 (56.7-64.5) | 15.5 (14.5-16.1) | 123.5 (116.0-126.0) |
| [Lao PDR.](http://www.who.int/countries/lao/en/) | 7,275,556 | 24.4 | 68.9 | 1.35 (1.19-1.97) | 37.0 (25.0-44.9) | 0.9 (0.7-1.1) | 29.0 (21.1-38.1) | 5.6 (3.9-6.8) | 255.0 (225.0-321.0) |
| [Malaysia](http://www.who.int/countries/mys/en/) | 32,365,998 | 30.3 | 76.7 | 1.55 (1.44-2.37) | 51.0 (42.2-58.9) | 1.7 (1.4-1.8) | 44.5 (35.3-49.6) | 8.3 (6.9-9.4) | 191.0 (182.0-204.0) |
| [Mongolia](http://www.who.int/countries/mng/en/) | 3,278,292 | 28.2 | 70.5 | 1.45 (1.31-2.16) | 43.0 (33.1-51.5) | 1.1 (0.9-1.3) | 37.0 (28.0-43.7) | 6.8 (5.2-7.8) | 192.5 (181.0-221.0) |
| [New Zealand](http://www.who.int/countries/nzl/en/) | 4,822,233 | 38.0 | 82.8 | 1.85 (1.73-2.82) | 63.5 (56.6-69.1) | 3.3 (3.1-3.7) | 51.5 (46.1-56.5) | 11.7 (10.6-12.6) | 125.9 (124.0-127.0) |
| [Pap. New Gui](http://www.who.int/countries/png/en/) | 8,947,027 | 22.4 | 65.2 | 1.25 (1.13-1.86) | 31.0 (20.2-40.3) | 0.8 (0.6-0.9) | 27.0 (17.1-34.2) | 5.0 (3.2-6.1) | 295.0 (254.0-409.0) |
| [Philippines](http://www.who.int/countries/phl/en/) | 109,581,085 | 25.7 | 71.7 | 1.35 (1.27-2.09) | 41.0 (30.4-48.9) | 1.2 (1.0-1.4) | 35.0 (25.5-41.3) | 6.5 (4.9-7.6) | 262.5 (239.0-308.0) |
| [Rep.Korea](http://www.who.int/countries/kor/en/) | 51,269,183 | 43.7 | 83.5 | 2.15 (1.93-3.15) | 73.5 (67.4-78.4) | 3.6 (3.4-4.0) | 59.5 (55.0-64.2) | 13.5 (12.4-14.2) | 124.5 (122.0-129.0) |
| Taiwan | 23,816,775 | 42.5 | 81.0 | 2.15 (1.91-3.12) | 73.5 (66.7-77.8) | 3.5 (3.3-3.9) | 59.5 (54.4-63.8) | 13.3 (12.2-14.0) | 120.3 (118.0-125.0) |
| [Singapore](http://www.who.int/countries/sgp/en/) | 5,850,343 | 42.2 | 84.1 | 2.05 (1.91-3.11) | 73.5 (66.9-78.3) | 3.2 (3.0-3.5) | 60.5 (54.9-64.5) | 13.1 (12.0-13.8) | 112.3 (109.0-115.0) |
| [Viet Nam](http://www.who.int/countries/vnm/en/) | 97,338,583 | 32.5 | 75.8 | 1.65 (1.52-2.50) | 55.0 (46.7-62.2) | 1.9 (1.7-2.1) | 47.5 (38.9-52.2) | 9.1 (7.8-10.1) | 185.5 (179.0-193.0) |
| WPRO | 1,960,061,147 | 37.9 | 77.5^c^ | 1.85 (1.25-2.30)^c^ | 63.5 (31.0-76.5)^c^ | 2.7 (0.8-5.3)^c^ | 51.5 (27.0-61.5)^c^ | 11.5 (5.0-15.5)^c^ | 170.9 (106.5-295.0)^c^ |

Lao PDR: [Lao People's Democratic Republic](http://www.who.int/countries/lao/en/); N: number; [Pap New Gui](http://www.who.int/countries/png/en/): [Papua New Guinea](http://www.who.int/countries/png/en/); [Rep Korea](http://www.who.int/countries/kor/en/): [Republic of Korea](http://www.who.int/countries/kor/en/); UI: uncertainty interval.

^a^For each epidemiologic indicator, the table reports the most probable estimate and the 95% uncertainty interval. For a number of countries, *R*_0_ was close to 1 in some of the uncertainty runs leading to an estimated time for peak incidence beyond the simulation duration of two years (delayed epidemic peak). For these countries, the upper limit of the uncertainty interval was set at 700 days, that is the end day of the simulation. Uncertainty runs in which *R*_0_ was less than 1 led to no epidemic emergence, and thus were excluded from further analysis. Reported ranges for the epidemic indicators did not include these runs, except for the range of *R*_0_.

^c^Estimates are for the median (and range).

**S4 Fig. Impact of the variation in median age in any country on the basic reproduction number, *R*_0_.** This figure was produced based on the estimates of all countries.

**
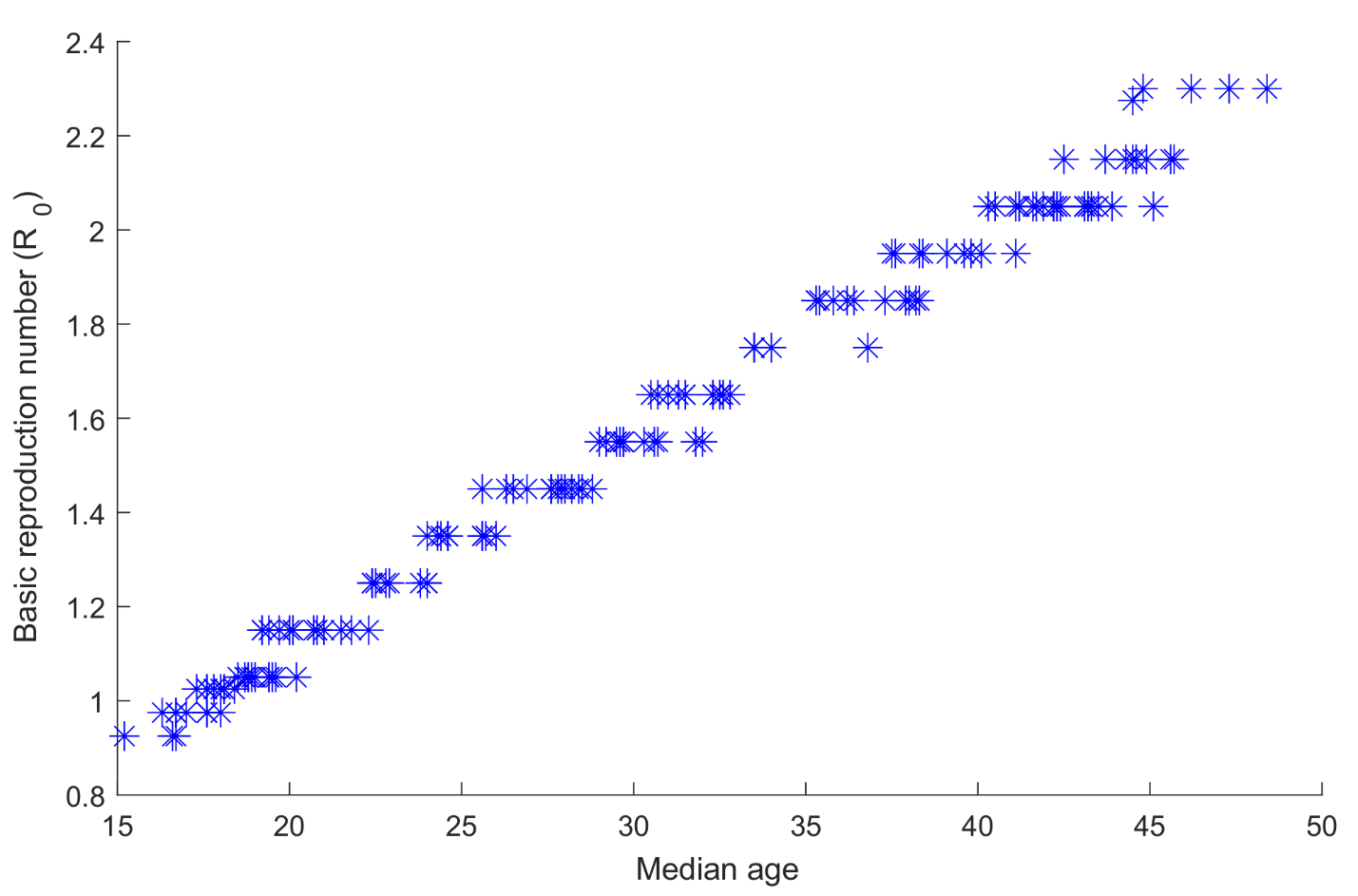
**

**S5 Fig. Age-specific cumulative incidence of the 2009 influenza A (H1N1) pandemic (H1N1pdm) virus.** This figure was reproduced from *Van Kerkhove et al.* [16].

**
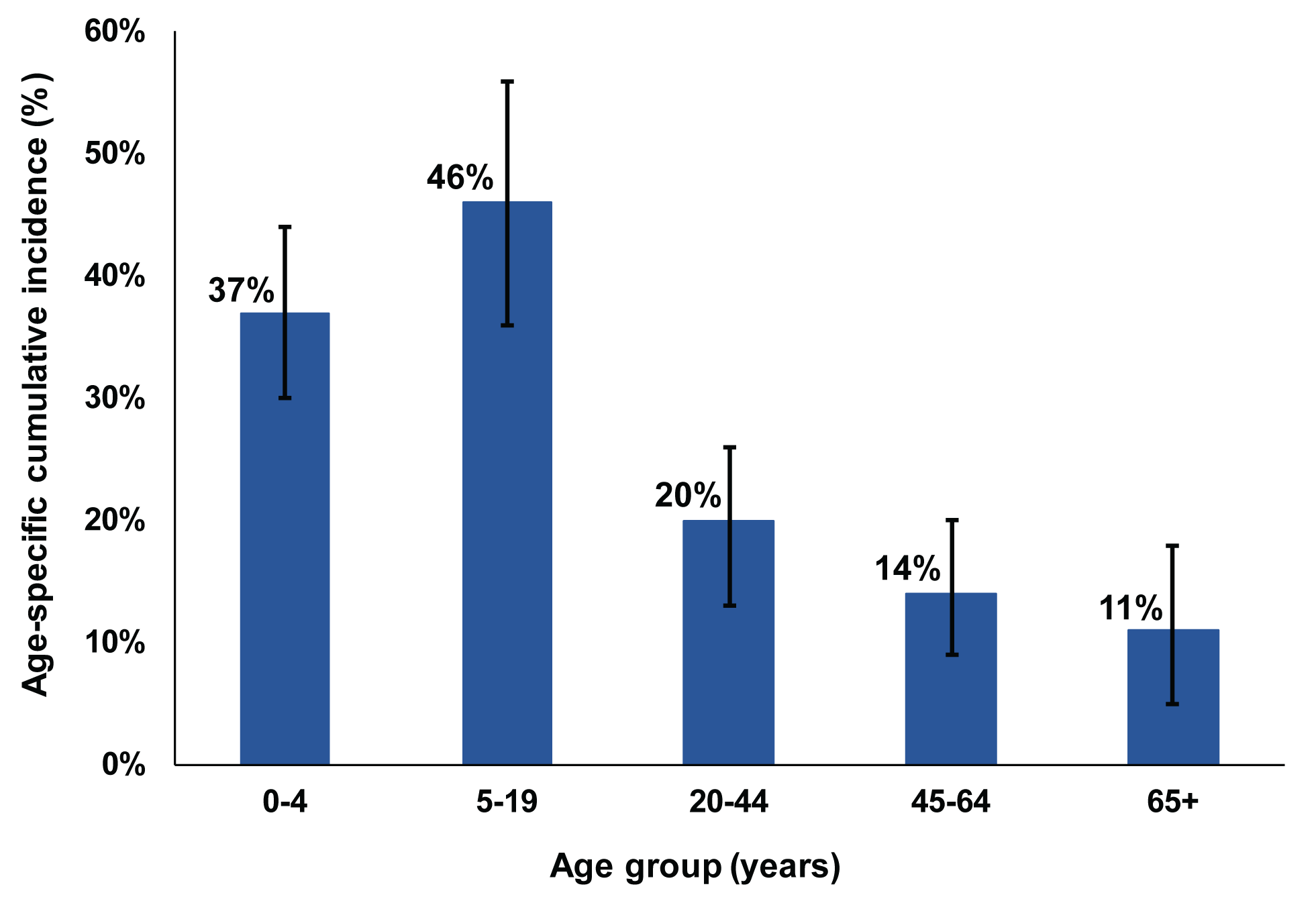
S6 Fig. Distribution of SARS-CoV-2 age-specific attack rate per 10,000 persons for Australia, Austria, Denmark, Finland, France, Germany, Iceland, and Italy [7, 17].
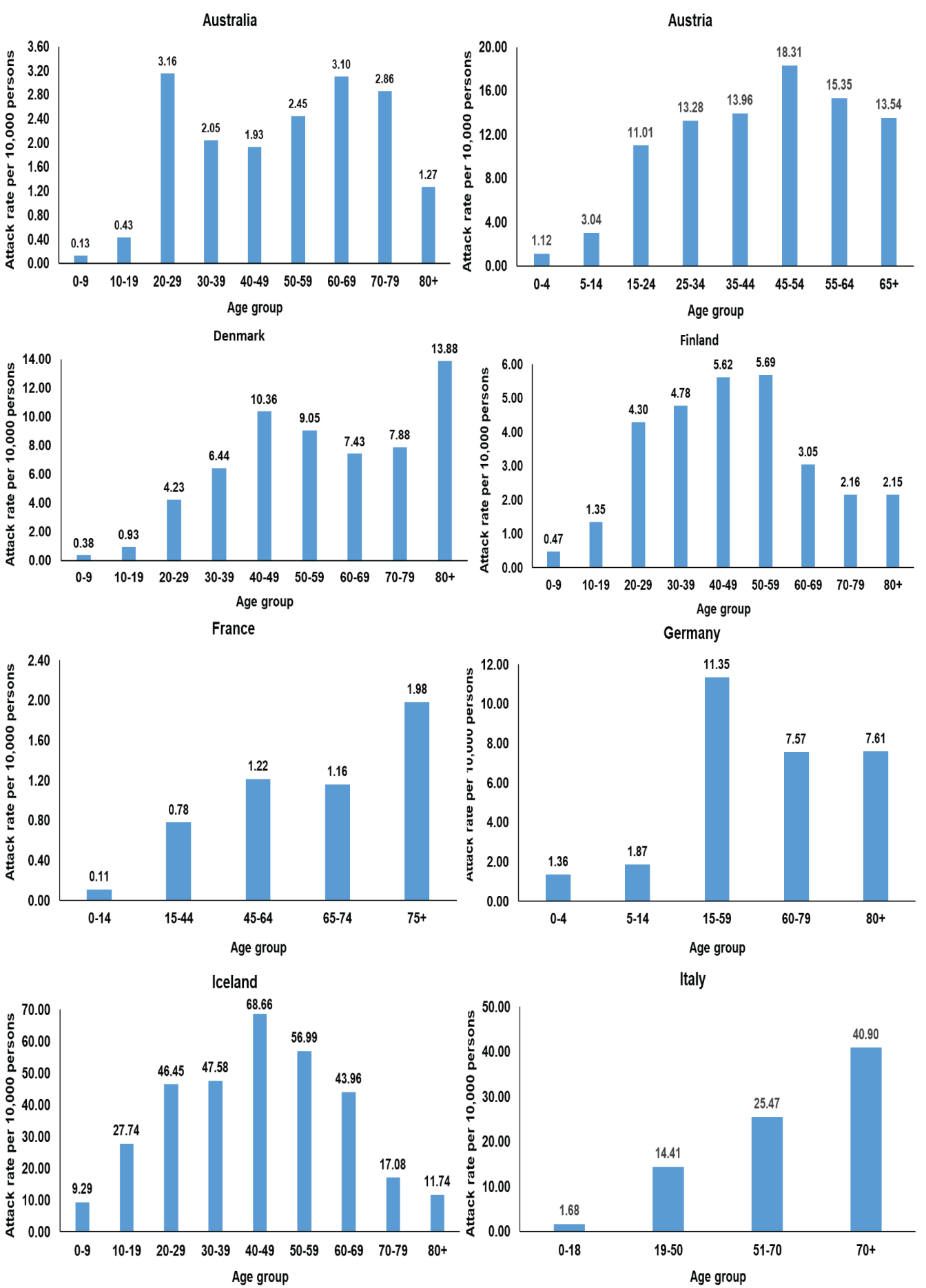
**

**S7 Fig. Distribution of SARS-CoV-2 age-specific attack rate per 10,000 persons for Japan, Norway, Singapore, Sweden, and Republic of Korea [7, 17].**

**
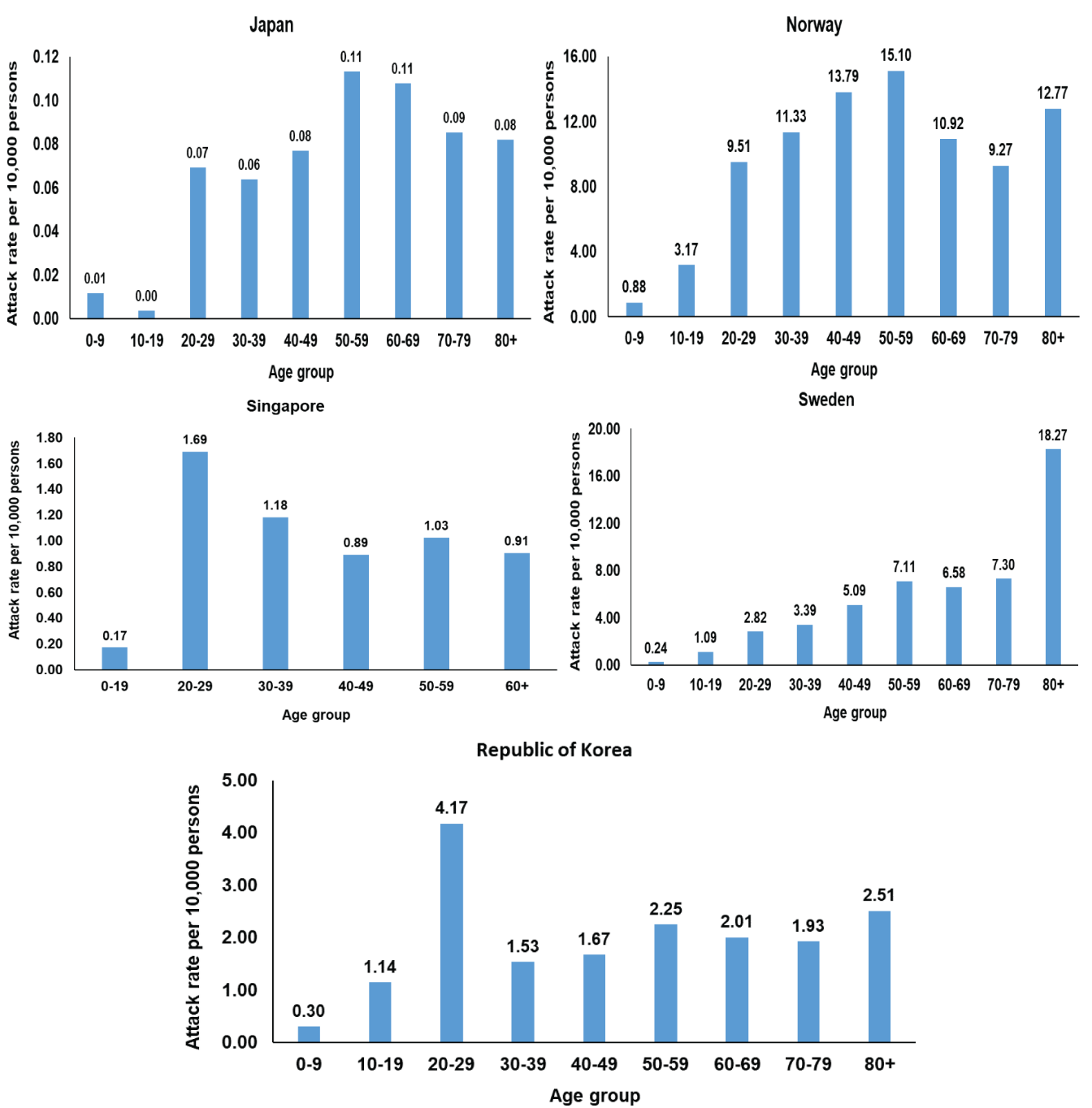
**

**S8 Fig. Sensitivity analysis assessing the impact of a 50% increase in the susceptibility to SARS-CoV-2 infection among those aged <30 years on our estimates for the basic reproduction number, *R*_0_, for the select countries presented in the main text.** The figure also includes, for comparison, estimates of *R*_0_ without assuming the 50% susceptibility enhancement.


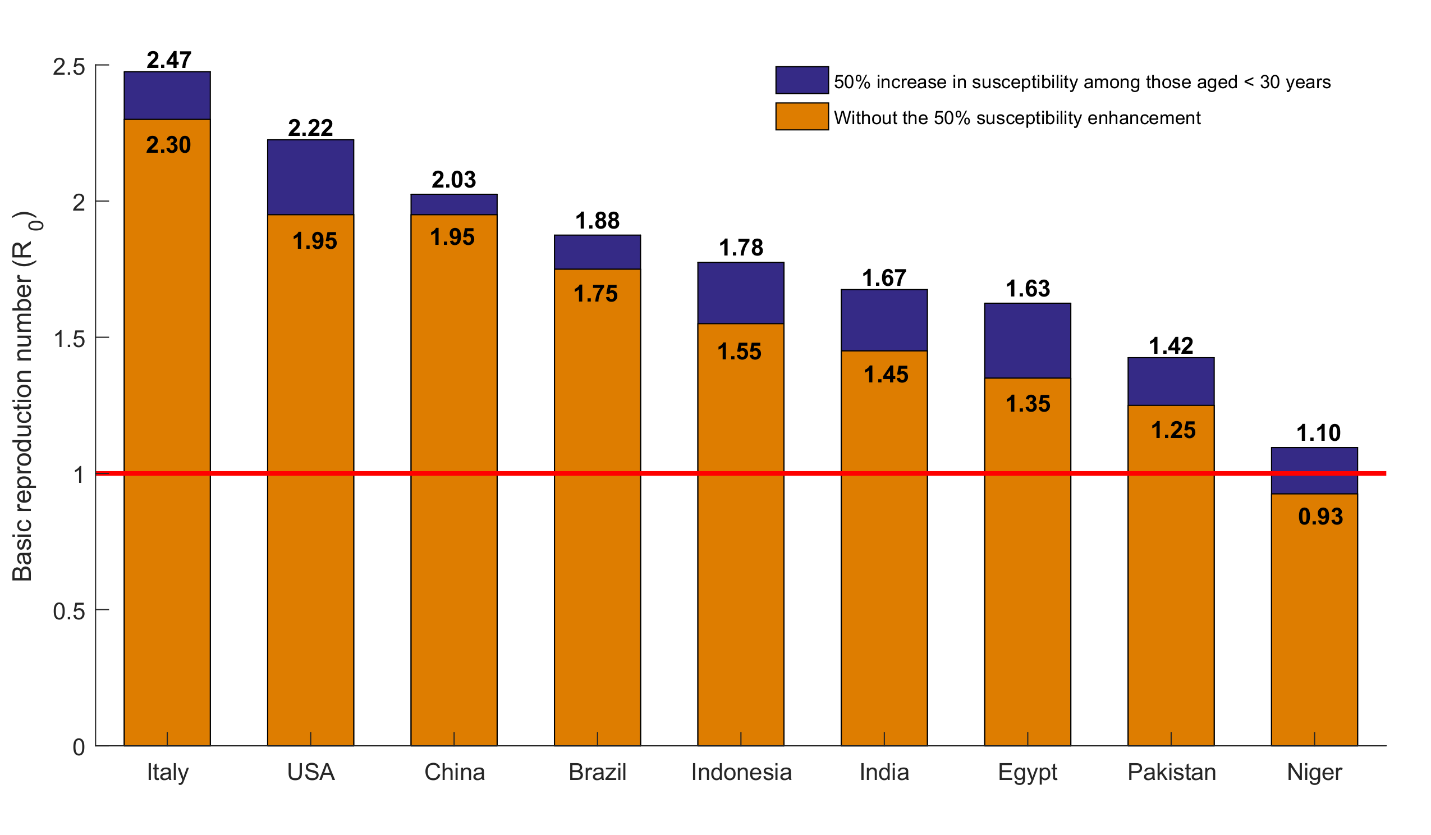


**S9 Fig. Sensitivity analysis assessing the impact of equal susceptibility to SARS-CoV-2 infection among those aged <20 to those aged 20-29 years on our estimates for the basic reproduction number, *R*_0_, for the select countries presented in the main text.** The figure also includes, for comparison, estimates of *R*_0_ without assuming this change in susceptibility profile.


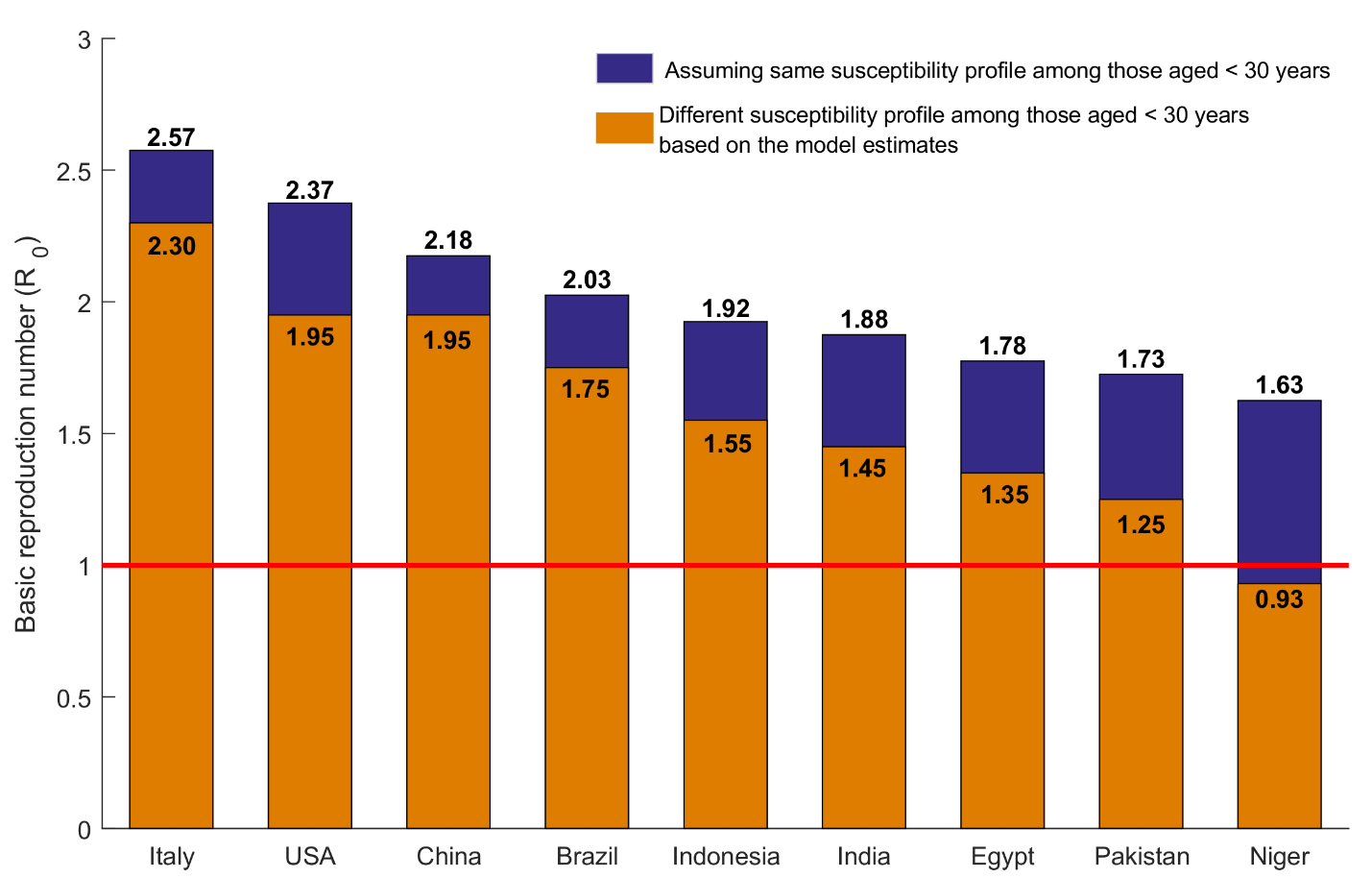


**S10 Fig. Sensitivity analysis assessing the impact of high degree of assortativeness in the age group mixing on our estimates for the basic reproduction number, *R*_0_, for the select countries presented in the main text.** The figure also includes, for comparison, estimates of *R*_0_ without assuming this change in the degree of assortativeness in age group mixing.

**
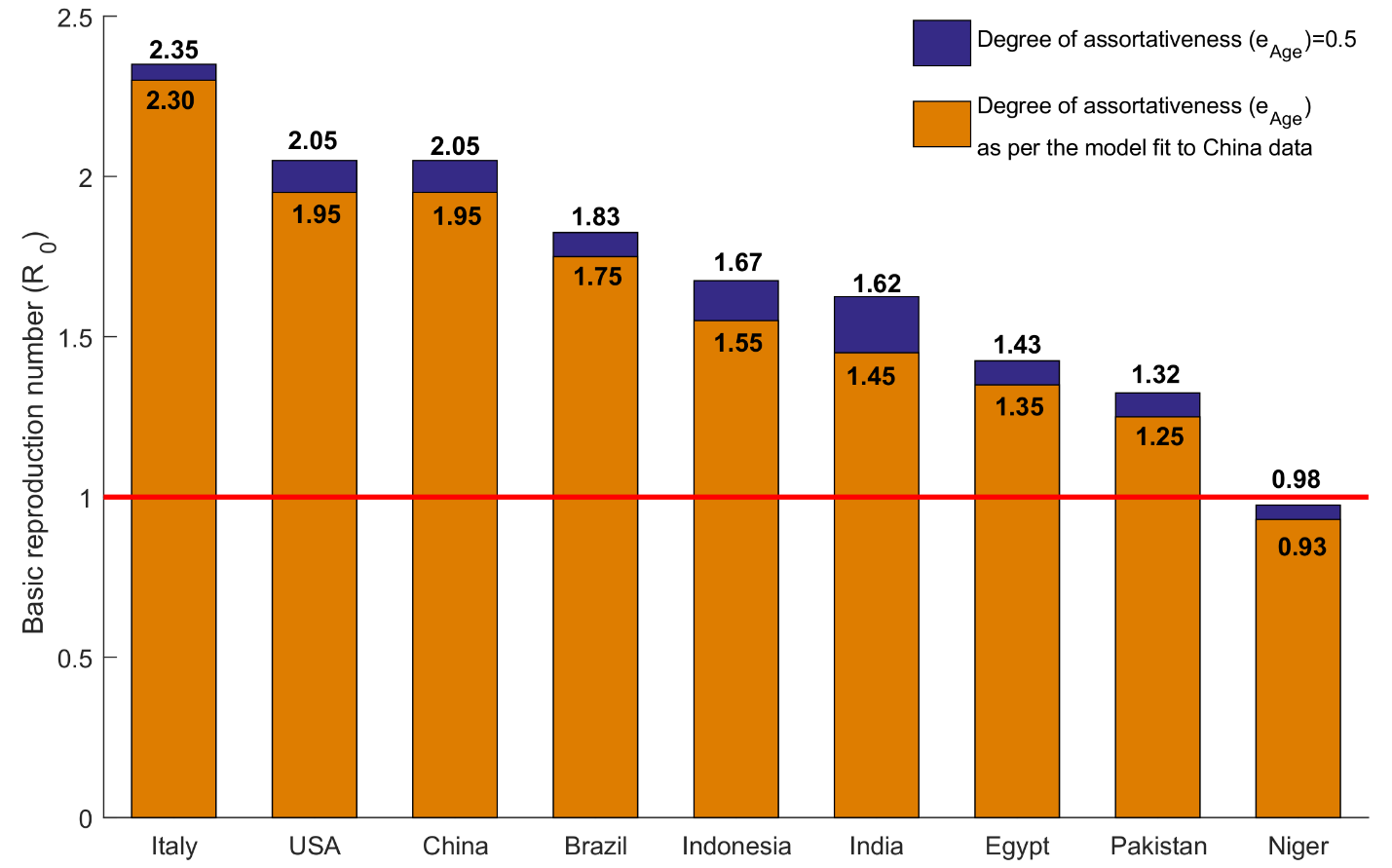
References**

1. Ayoub HH, Chemaitelly H, Mumtaz GR, Seedat S, Awad SF, Makhoul M, et al. Characterizing key attributes of the epidemiology of COVID-19 in China: Model-based estimations. medRxiv. 2020:2020.04.08.20058214. doi: 10.1101/2020.04.08.20058214.

2. Guan WJ, Ni ZY, Hu Y, Liang WH, Ou CQ, He JX, et al. Clinical Characteristics of Coronavirus Disease 2019 in China. N Engl J Med. 2020. doi: 10.1056/NEJMoa2002032. PubMed PMID: 32109013.

3. Huang C, Wang Y, Li X, Ren L, Zhao J, Hu Y, et al. Clinical features of patients infected with 2019 novel coronavirus in Wuhan, China. Lancet. 2020;395(10223):497-506. doi: 10.1016/S0140-6736(20)30183-5. PubMed PMID: 31986264.

4. World Health Organization. Report of the WHO-China Joint Mission on Coronavirus Disease 2019 (COVID-19). Available from :<https://www.who.int/docs/default-source/coronaviruse/who-china-joint-mission-on-covid-19-final-report.pdf>. Accessed on March 10, 2020. 2020.

5. Novel Coronavirus Pneumonia Emergency Response Epidemiology Team. [The epidemiological characteristics of an outbreak of 2019 novel coronavirus diseases (COVID-19) in China]. Zhonghua Liu Xing Bing Xue Za Zhi. 2020;41(2):145-51. doi: 10.3760/cma.j.issn.0254-6450.2020.02.003. PubMed PMID: 32064853.

6. Wu Z, McGoogan JM. Characteristics of and Important Lessons From the Coronavirus Disease 2019 (COVID-19) Outbreak in China: Summary of a Report of 72314 Cases From the Chinese Center for Disease Control and Prevention. JAMA. 2020. doi: 10.1001/jama.2020.2648. PubMed PMID: 32091533.

7. United Nations Department of Economic and Social Affairs Population Dynamics. The 2019 Revision of World Population Prospects. Available from <https://population.un.org/wpp/>. Accessed on March 1st, 2020. 2020.

8. Li R, Pei S, Chen B, Song Y, Zhang T, Yang W, et al. Substantial undocumented infection facilitates the rapid dissemination of novel coronavirus (SARS-CoV2). Science. 2020;368(6490):489-93. doi: 10.1126/science.abb3221. PubMed PMID: 32179701.

9. Lauer SA, Grantz KH, Bi Q, Jones FK, Zheng Q, Meredith HR, et al. The Incubation Period of Coronavirus Disease 2019 (COVID-19) From Publicly Reported Confirmed Cases: Estimation and Application. Ann Intern Med. 2020;172(9):577-82. doi: 10.7326/M20-0504. PubMed PMID: 32150748.

10. Zou L, Ruan F, Huang M, Liang L, Huang H, Hong Z, et al. SARS-CoV-2 Viral Load in Upper Respiratory Specimens of Infected Patients. N Engl J Med. 2020;382(12):1177-9. doi: 10.1056/NEJMc2001737. PubMed PMID: 32074444.

11. Rothe C, Schunk M, Sothmann P, Bretzel G, Froeschl G, Wallrauch C, et al. Transmission of 2019-nCoV Infection from an Asymptomatic Contact in Germany. N Engl J Med. 2020;382(10):970-1. doi: 10.1056/NEJMc2001468. PubMed PMID: 32003551.

12. Shekerdemian LS, Mahmood NR, Wolfe KK, Riggs BJ, Ross CE, McKiernan CA, et al. Characteristics and Outcomes of Children With Coronavirus Disease 2019 (COVID-19) Infection Admitted to US and Canadian Pediatric Intensive Care Units. JAMA Pediatr. 2020. doi: 10.1001/jamapediatrics.2020.1948. PubMed PMID: 32392288.

13. Team CC-R. Coronavirus Disease 2019 in Children - United States, February 12-April 2, 2020. MMWR Morb Mortal Wkly Rep. 2020;69(14):422-6. doi: 10.15585/mmwr.mm6914e4. PubMed PMID: 32271728; PubMed Central PMCID: PMCPMC7147903 Journal Editors form for disclosure of potential conflicts of interest. No potential conflicts of interest were disclosed.

14. Dong Y MX, Hu Y, Qi X, Jiang F, Jiang Z, Tong S,. Epidemiological Characteristics of 2143 Pediatric Patients With 2019 Coronavirus Disease in China. Pediatrics. 2020:e20200702. doi: doi:10.1542/peds.2020-0702.

15. Heffernan JM, Smith RJ, Wahl LM. Perspectives on the basic reproductive ratio. Journal of the Royal Society Interface. 2005;2(4):281-93.

16. Van Kerkhove MD, Hirve S, Koukounari A, Mounts AW, group HNpsw. Estimating age-specific cumulative incidence for the 2009 influenza pandemic: a meta-analysis of A(H1N1)pdm09 serological studies from 19 countries. Influenza Other Respir Viruses. 2013;7(5):872-86. doi: 10.1111/irv.12074. PubMed PMID: 23331969; PubMed Central PMCID: PMCPMC5781221.

17. Statista. Number of coronavirus (COVID-19) cases by country. Available from: <https://www.statista.com/statistics/1043366/novel-coronavirus-2019ncov-cases-worldwide-by-country/> Accessed on April 15, 2020. 2020.
